# Lung tumors with distinct p53 mutations respond similarly to p53 targeted therapy but exhibit genotype-specific statin sensitivity

**DOI:** 10.1101/gad.298463.117

**Published:** 2017-07-01

**Authors:** Frances K. Turrell, Emma M. Kerr, Meiling Gao, Hannah Thorpe, Gary J. Doherty, Jake Cridge, David Shorthouse, Alyson Speed, Shamith Samarajiwa, Benjamin A. Hall, Meryl Griffiths, Carla P. Martins

**Affiliations:** 1 Medical Research Council (MRC) Cancer Unit, University of Cambridge, Hutchison/MRC Research Centre, Cambridge CB2 0XZ, United Kingdom;; 2 Department of Oncology, Addenbrooke's Hospital, Cambridge, CB2 0QQ, United Kingdom;; 3 Department of Histopathology, Cambridge University Hospitals National Health Service Foundation Trust, Cambridge, CB2 0QQ, United Kingdom

**Keywords:** mutant p53, lung tumors, transcriptome analysis, p53 restoration, mevalonate pathway, therapy

## Abstract

Lung adenocarcinoma accounts for ∼40% of lung cancers, the leading cause of cancer-related death worldwide, and current therapies provide only limited survival benefit. Approximately half of lung adenocarcinomas harbor mutations in *TP53* (p53), making these mutants appealing targets for lung cancer therapy. As mutant p53 remains untargetable, mutant p53-dependent phenotypes represent alternative targeting opportunities, but the prevalence and therapeutic relevance of such effects (gain of function and dominant-negative activity) in lung adenocarcinoma are unclear. Through transcriptional and functional analysis of murine *Kras*^*G12D*^-*p53*^*null*^, -*p53*^*R172H*^ (conformational), and -*p53*^*R270H*^ (contact) mutant lung tumors, we identified genotype-independent and genotype-dependent therapeutic sensitivities. Unexpectedly, we found that wild-type p53 exerts a dominant tumor-suppressive effect on mutant tumors, as all genotypes were similarly sensitive to its restoration in vivo. These data show that the potential of p53 targeted therapies is comparable across all p53-deficient genotypes and may explain the high incidence of p53 loss of heterozygosity in mutant tumors. In contrast, mutant p53 gain of function and their associated vulnerabilities can vary according to mutation type. Notably, we identified a *p53*^*R270H*^-specific sensitivity to simvastatin in lung tumors, and the transcriptional signature that underlies this sensitivity was also present in human lung tumors, indicating that this therapeutic approach may be clinically relevant.

## Abstract (precis)

In this study, Turrell et al. perform a comprehensive transcriptional and functional analysis of murine lung tumors with distinct p53 alterations (p53 loss, DNA contact [R270H], or conformational [R172H] mutations) and identified both common therapeutic vulnerabilities and mutation-specific liabilities in these tumors. Overall, their findings provide insight into new therapeutic approaches that may be clinically relevant for patients with mutant p53 lung tumors.

The p53 transcription factor is frequently counterselected during tumor development due to its ability to trigger a multitude of tumor-suppressive effects in response to a wide variety of cellular stress signals, including DNA damage and oncogene activation (Freed-Pastor and Prives 2012; Bieging et al. 2014). p53 mutations are present in ∼45% of lung adenocarcinomas and correlate with reduced survival (The Cancer Genome Atlas Research Network 2014). Most are missense mutations in the p53 DNA-binding region that can be classified as either contact (interfere directly with DNA binding) or conformational (induce local or global conformational distortions) mutations. Both groups affect the DNA-binding ability of p53 and are thus associated with loss of wild-type function (Joerger and Fersht 2007).

Mutant p53 is typically highly expressed in tumors, likely reflecting the presence of p53-inducing stress signals and the inability of the mutants to transcriptionally activate the p53-negative regulator Mdm2 (Freed-Pastor and Prives 2012). Due to their high prevalence and tumor specificity, p53 mutants are an attractive target for lung cancer therapy. As mutant p53 remains untargetable, the identification of mutant p53 tumor dependencies provides alternative targeting opportunities. Mutant p53 gain-of-function (GOF) phenotypes have been described in different tissues (Lang et al. 2004; Olive et al. 2004) and helped uncover potential targetable vulnerabilities in p53 mutant breast cancer (Freed-Pastor et al. 2012) and pancreatic ductal adenocarcinoma (PDAC) (Weissmueller et al. 2014). However, it remains unclear to what extent mutant p53 effects may be tissue-specific and even mutant type-specific and, in particular, whether mutant p53 GOFs are present in lung tumors. p53 targeted therapies, which aim to restore wild-type p53 activity in tumors where the protein is lost, mutated, or inhibited, represent another appealing strategy for the treatment of p53-deficient tumors (Cheok et al. 2011; Frezza and Martins 2012). However, given that p53 mutants can exert dominant-negative (DN) effects (Freed-Pastor and Prives 2012), the efficacy of wild-type p53 restoration therapies in mutant tumors is expected to be limited. Despite this, its potential impact on mutant p53 lung tumors has not yet been addressed. Interestingly, p53 mutant tumors, including lung adenocarcinomas, often display loss of heterozygosity (LOH) (Baker et al. 1990; Mitsudomi et al. 2000; Zienolddiny et al. 2001; Liu et al. 2016), suggesting that wild-type p53 activity may be counterselected even in the presence of the mutant. Hence, the extent of DN effects of mutant p53 in vivo and, conversely, those of wild-type p53 in mutant lung tumors remain unclear.

To identify potential therapeutic vulnerabilities in mutant p53 lung tumors, we characterized the transcriptional and functional phenotypes of murine tumors that lack p53 or express a contact (R270H, corresponding to R273H in humans) or conformational (R172H, corresponding to human R175H) p53 mutant. These tumors were analyzed in the presence or absence of wild-type p53 functionality (Christophorou et al. 2005) to identify potential DN and GOF mutant p53 phenotypes, respectively. Our study provides the first comprehensive characterization of wild-type p53, p53-null, and (conformational and contact) mutant p53 transcriptional signatures and their functional consequences in vivo. Importantly, these analyses also expose key therapeutic vulnerabilities of mutant Kras lung tumors with distinct p53 deficiencies.

## Results

### Generation of *Kras^G12D^;p53* mutant and *p53*-null lung tumor models for comparative transcriptional analysis

**Figure 1.**
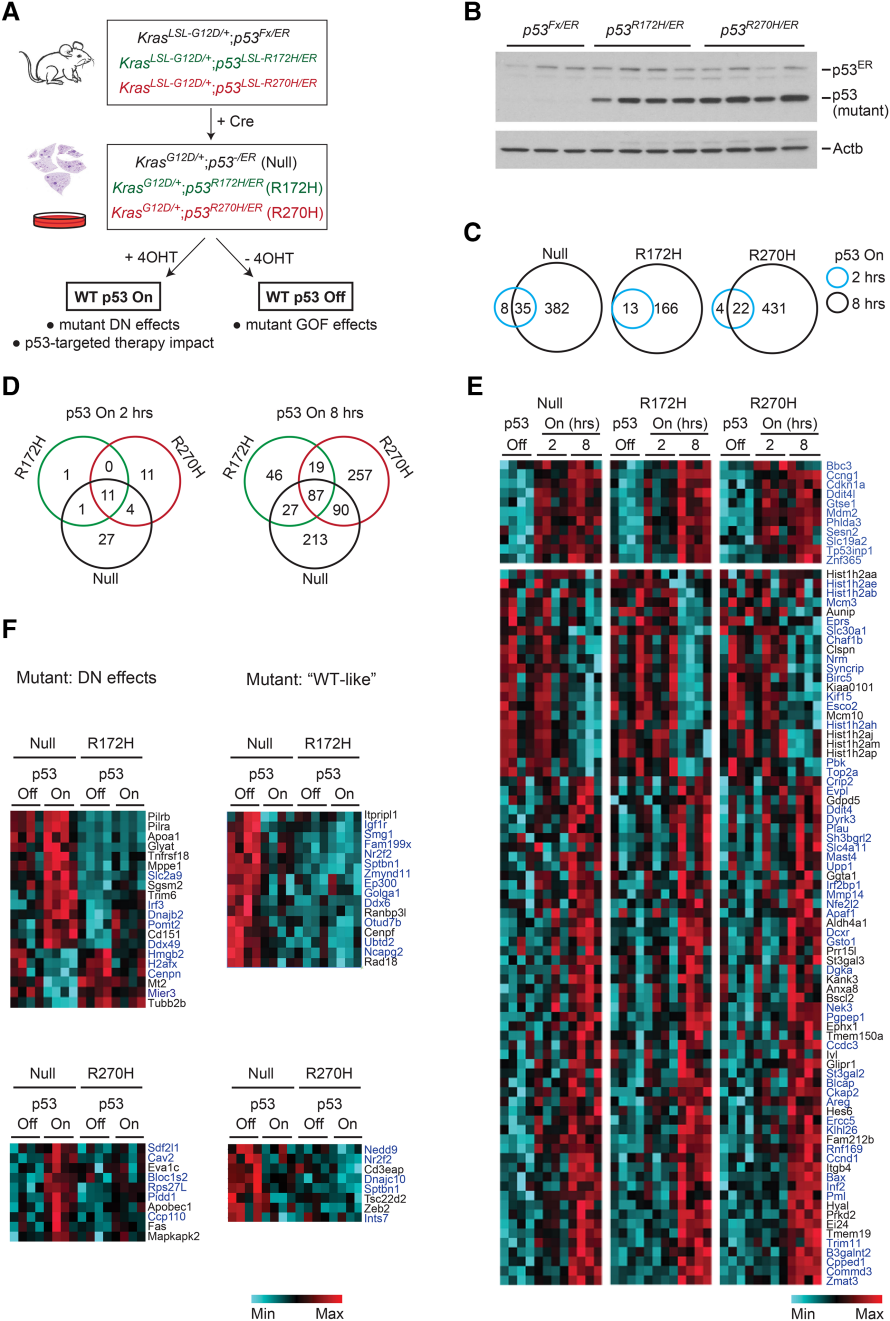
Transcriptome analysis of the effects of wild-type p53 on p53-null and mutant lung tumor cells. (*A*) Schematic representation of lung tumor models used in vitro and in vivo (p53^ER^ models). (*B*) Expression levels of (mutant) p53, p53^ER^, and Actb (loading control) in murine lung tumor cell lines of the indicated genotypes (immunoblotting). (*C*–*F*) Microarray analysis of null, R172H, and R270H lung tumor cell lines. Four independent cell lines per genotype were analyzed in the absence (off) or presence of functional p53 for 2 or 8 h (4OHT treatment; on). (*C*) Venn diagrams showing the number of genes differentially expressed relative to vehicle treatment in null, R172H, and R270H cells 2 h (blue) and 8 h (black) after p53 restoration. (*D*) Comparative analysis of transcriptional responses between genotypes 2 h (*left* panel) or 8 h (*right* panel) after 4OHT treatment. (*E*) Heat map showing the expression of genes similarly altered in null, R172H, and R270H cells upon 4OHT treatment. (*Top* panel) Eleven genes were induced (>1.4-fold) 2 h after treatment. (*Bottom* panels) The remaining genes were similarly altered in all genotypes 8 h after treatment. Known p53 targets are shown in blue (see Supplemental Table S1). (*F*) Heat maps showing examples of transcriptional DN effects (*left*) or “wild-type-like” (*right*) mutant p53 transcriptional phenotypes.

Lung adenocarcinoma can be recapitulated in mice through the conditional activation of two genetic alterations frequently coselected in the human disease; namely, oncogenic activation of *KRAS* and p53 inactivation (Jackson et al. 2005; The Cancer Genome Atlas Research Network 2014). To characterize the effects of p53 mutants in lung adenocarcinoma, we generated *Kras*^G12D^-driven murine tumors that either lack p53 (*p53*^*Fx*^ allele; i.e., p53^−^) or carry a contact (*p53^R270H^*) or conformational (*p53^R172H^*) mutation (Jonkers et al. 2001; Jackson et al. 2005). In addition, all models contained a second modified *p53* knock-in allele (*p53*^ER^) encoding the switchable p53^ER^ fusion protein, which enables the functionality of this endogenously expressed p53 to be controlled through the administration (p53 “on” state) or withdrawal (p53 “off” state) of 4-hydroxytamoxifen (4OHT). In the absence of 4OHT, p53^ER^ is equivalent to a knockout, while 4OHT administration restores wild-type function in vitro and in vivo (Christophorou et al. 2005; Junttila et al. 2010). The following tumor models were generated: (1) *Kras*^G12D/+^*;p53*^−/ER^, (2) *Kras*^G12D/+^*;p53*^R172H/ER^, and (3) *Kras*^G12D/+^*;p53*^R270H/ER^ (Fig. 1A). For simplicity, they are referred to here as “null,” “R172H,” and “R270H,” respectively. Similar models with the *p53*^*ER*^ allele replaced by *p53*^*Fx*^ were also developed and are mentioned specifically where relevant.

Lung tumors were induced as described previously (Junttila et al. 2010; Kerr et al. 2016), and multiple lung tumor cell lines were generated per genotype, each from an independent tumor never exposed to 4OHT. Using a differential attachment method, the presence of tumor-associated fibroblasts (TAFs) in tumor cell cultures was limited to <8% (Supplemental Fig. S1A). All tumor cell lines showed full recombination of the relevant alleles, but, interestingly, none of these was recombined in TAF cultures (Supplemental Fig. S1B; data not shown). Genotypes were confirmed through PCR and sequencing (Supplemental Fig. S1C; data not shown). As expected, both mutants were expressed at significantly higher levels than the wild-type protein (p53^ER^) in mutant cells (Fig. 1B).

To compare the transcriptional signatures of null, R172H, and R270H lung tumor cells, four independent cell lines per genotype cultured in Matrigel were analyzed by microarray. Cells were cultured with and without wild-type p53 restoration to assess for DN and GOF effects of the mutants, respectively (Fig. 1A). Differential expression was defined as a change in gene expression of >1.4-fold between cohorts. To compensate for intragenotype variation effects, only genes similarly deregulated across all cell lines of a given genotype were considered in the microarray analyses presented (intragenotype variation: SD < 0.5 across four biological replicates).

### ‘Canonical’ p53 target genes are efficiently induced by wild-type p53 in p53 mutant lung tumor cells

To identify potential DN effects of the R172H and R270H p53 mutants in lung tumors and simultaneously determine the effect of p53 targeted therapy on these tumors, we assessed the transcriptional impact of wild-type p53 on p53-null and p53 mutant cells. Three-dimensional (3D) cultures of p53-null, R172H, and R270H (*n* = 4 per genotype) were treated with control vehicle or 4OHT, and their transcriptional profiles were analyzed. Both immediate (2 h after 4OHT treatment) and sustained (8 h after 4OHT) p53-mediated transcriptional responses were assessed.

Restoration of p53 functionality significantly altered gene expression in all cell lines. Two hours after p53 restoration, a small number of genes was up-regulated/down-regulated within each genotype (*p53^null^*: 43 genes; *p53^R172H^*: 13 genes; *p53^R270H^* 26 genes) (Fig. 1C). Eleven known p53 targets (*Mdm2*, *Cdkn1a* [*p21*], *Bbc3* [*Puma*]*, Ddit4l*, *Zfp365*, *Phlda3*, *Trp53inp*, *Gtse1*, *Ccng1*, *Sesn2*, and *Slc19a2*) (Utrera et al. 1998; Lo et al. 2001; Bieging et al. 2014; Quintens et al. 2015) were similarly regulated across genotypes (Fig. 1D [left], E [top]). Given their early induction, these are likely direct p53 targets in lung tumor cells. In agreement, nine of these genes (82%) were identified independently as direct p53 targets through ChIP-seq (chromatin immunoprecipitation [ChIP] combined with high-throughput sequencing) analysis (Supplemental Tables S1, S2; Fischer 2017; http://chip-atlas.org), indicating that the “immediate” p53-responsive genes in lung tumor cells are likely direct p53 targets in different cellular contexts. Importantly, since “core” p53 target genes such as *Mdm2* and *p21* were efficiently and promptly induced by p53 restoration in the presence of endogenously expressed mutant p53, our data argue against a DN effect of the mutants regarding these “canonical” p53 targets.

Eight hours after treatment, the number of p53-regulated genes was significantly increased (p53^null^: 417 genes; p53^R172H^: 179 genes; p53^R270H^: 453 genes) (Fig. 1C). Strikingly, 87 genes (including the common 2-h subset) were similarly regulated by p53 across all genotypes (Fig. 1D [right], E). Pathway analysis identified “p53 signaling” as the top canonical pathway similarly regulated across genotypes (data not shown). Accordingly, most similarly regulated genes are well-established p53 targets (i.e., “canonical” targets) (Fig. 1E, blue; Supplemental Table S1, S2). As seen for the 2-h cohort, the majority of genes similarly regulated in all genotypes 8 h after treatment (64%) is directly bound by p53 in other contexts, suggesting that these genes are direct p53 targets even if they are not immediate ones. Among these were genes involved in different p53-mediated responses, such as cell cycle arrest, apoptosis, DNA repair, autophagy, and senescence (Supplemental Fig. S1D). Hence, wild-type p53 is able to retain a significant part of its transcriptional activity in mutant p53 lung tumor cells, including the induction of some of its key targets, providing a potential explanation for the high frequency of p53 LOH in mutant tumors (Baker et al. 1990; Mitsudomi et al. 2000; Zienolddiny et al. 2001; Liu et al. 2016).

### p53 mutants exert DN and wild-type-like transcriptional effects in lung tumor cells

While p53 restoration had similar effects on the expression of key p53 target genes across all genotypes, genotype-specific transcriptional signatures were nevertheless observed, particularly at the 8-h time point (Fig. 1D). Accordingly, 213 out of the 417 genes induced by wild-type p53 in null cells were not significantly altered in mutant lines, providing evidence of mutant DN activity. However, the extent of these DN effects was variable, spanning from complete failure (R172H: 72 genes, R270H: 27 genes) to reduced ability of wild-type p53 to induce/repress its targets in mutant cells (1.2 < fold change < 1.4; R172H = 143 genes; R270H = 155 genes). Interestingly, a subset of genes (R172H: 87 genes; R270H: 43 genes) showed levels of expression in mutant cells (p53 off) similar to those seen in null upon p53 restoration, suggesting that mutant proteins retain wild-type p53 transcriptional activity (Fig. 1F; data not shown). Analysis of ChIP-seq data sets suggests that the majority of genes included in these “wild-type-like” signatures can be directly bound by p53 (∼70%), while genes included in DN signatures are less likely to be direct p53 targets (41%–55%) (Supplemental Table S2).

Collectively, our microarray analysis revealed that endogenous expression of wild-type p53 in p53 mutant R172H and R270H lung tumor cells triggers a complex transcriptional response involving both wild-type tumor-suppressive activity and DN signatures. Interestingly, the majority of the DN phenotypes observed appears to be partial, as the corresponding p53 target genes are still regulated by the wild type in mutant cells, albeit to a lower degree.

### Endogenously expressed wild-type p53 induces cell cycle arrest and cell death in R172H and R270H mutant lung tumor cells

**Figure 2.**
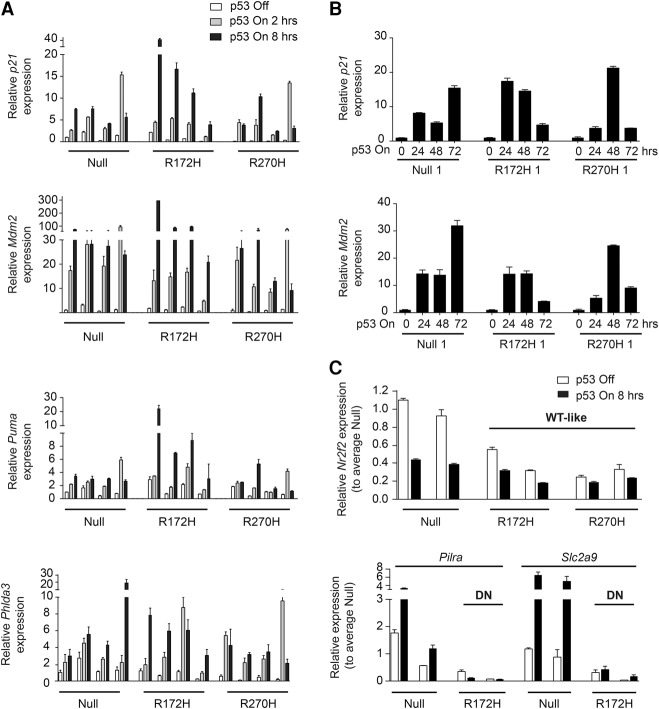
Regulation of expression of known p53 targets by wild-type p53 in p53-null and mutant lung tumor cells. Representative TaqMan data of two independent runs showing expression of p53 targets in null, R172H, and R270H cell lines. (*A*) Expression of the indicated genes 2 and 8 h after p53 restoration (p53 on) relative to the vehicle-treated (p53 off) null cell line (first bar). Four cell lines per genotype are shown (see Supplemental Fig. S2B). (*B*) p53 target gene expression 24, 48, and 72 h after p53 restoration relative to vehicle at the corresponding time points. One cell line per genotype is shown (see Supplemental Fig. S2C). (*C*) Expression profiling of genes differentially expressed between p53-null and the indicated mutant cells. Two cell lines per genotype are shown. Values are shown relative to the average of the corresponding null samples. (*A*–*C*) The SD of the triplicate mean per cell line is shown.

To confirm the transcriptional activity of wild-type p53 in mutant cells, we examined the expression of p53 target genes in all 12 cell lines by TaqMan analysis. No difference in the expression of typical “canonical” p53 targets (e.g., *Mdm2*, *p21*, and *Puma*) was observed between genotypes in the absence of treatment (Supplemental Fig. S2A). p53 restoration resulted in a significant up-regulation of typical p53 targets in all genotypes 2 and 8 h after treatment (Fig. 2A; Supplemental Fig. S2B), confirming their responsiveness to wild-type p53 in mutant cells. A high degree of variation was observed between cell lines regarding timing and fold of target induction, but, in most cases, gene induction increased over time (2 h < 8 h time point). Expression profiling of later time points revealed that p53 targets remained induced up to 72 h after treatment, but the levels of expression varied significantly between cell lines both between and within genotypes (Fig. 2B; Supplemental Fig. S2C). No clear genotype-specific response pattern could be detected 72 h after p53 restoration, but target gene expression was frequently higher in the null cells. DN and wild-type-like expression profiles of mutant cells were also confirmed by TaqMan analysis (Fig. 2C; data not shown).

**Figure 3.**
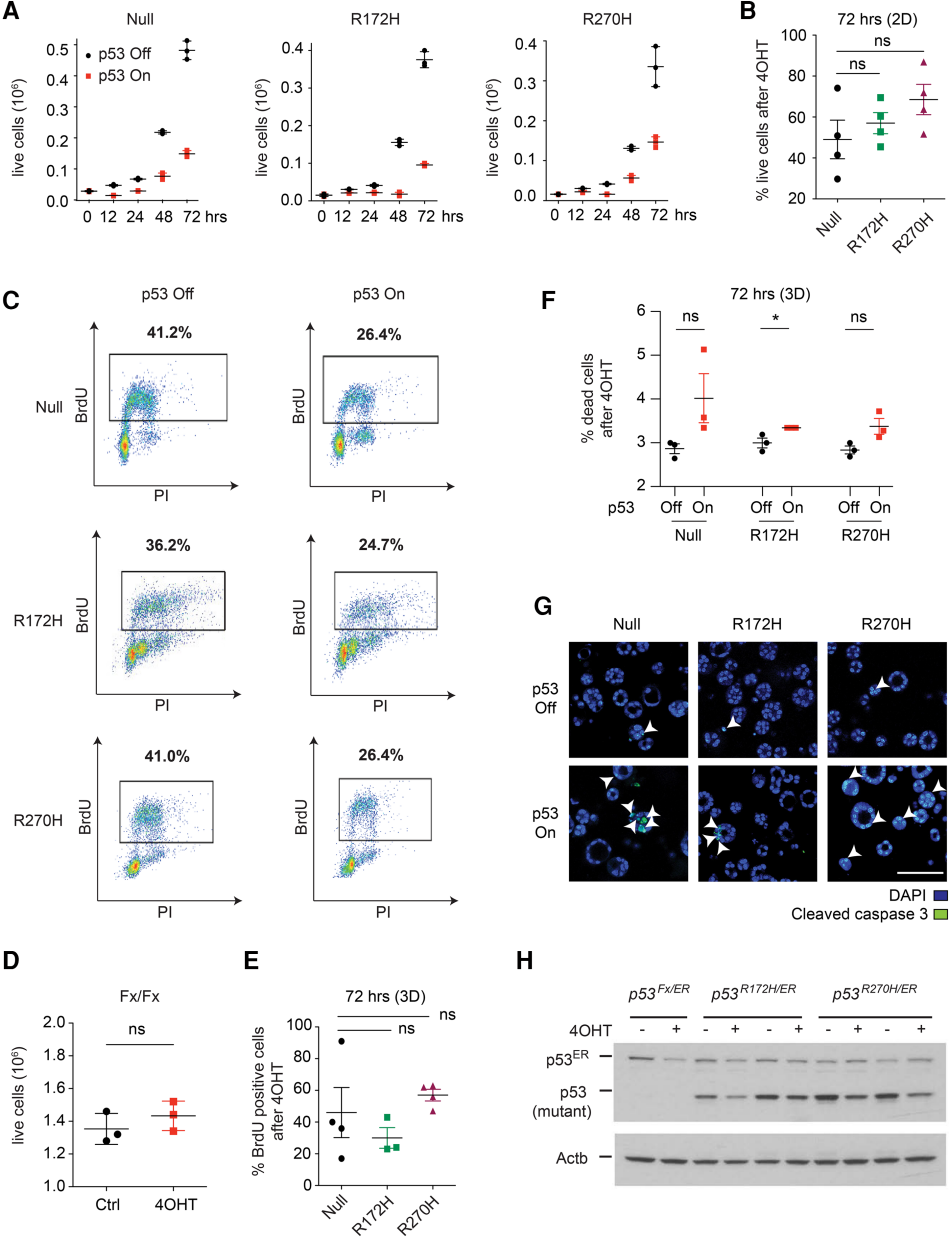
Wild-type p53 induces similar cell cycle arrest and apoptotic responses in p53-null, R172H, and R270H lung tumor cells. (*A*) Growth curves of null (*left*), R172H (*middle*), and R270H (*right*) cells following vehicle (p53 off) or 4OHT (p53 on) treatment. One representative cell line per genotype is shown (see Supplemental Fig. S3A). *n* = 4. Circles depict technical triplicates, and the average ± SD is indicated. (*B*) Viability of cells of the indicated genotypes 72 h after p53 restoration, relative to vehicle. Four independent cell lines per genotype are shown ±SEM. (*C*) Representative (*n* = 4 per genotype) BrdU/PI analysis (FACS) of the indicated cells 24 h after vehicle or 4OHT treatment. The percentage of cells in S phase is indicated. (*D*) Viability of *Kras*^*G12D*^;*p53*^*Fx/Fx*^ murine lung tumor cell lines 72 h after 4OHT/Ctrl treatment. One representative cell line is shown. *n* = 3. Circles represent technical replicates, and the mean ± SD is shown. (*E*) The percentage of BrdU-positive cells (FACS) in null, R172H, and R270H cultures 72 h after 4OHT treatment, relative to vehicle. Symbols denote independent cell lines. SEM of the average percentage per genotype is indicated. (*F*) Representative data (*n* = 3) showing the percentage of dead cells for one cell line per genotype 72 h after vehicle/4OHT treatment. (*G*) Representative images of cleaved caspase 3 staining of null, R172H, and R270H cells 72 h after vehicle/4OHT treatment. Arrowheads indicate examples of positive staining. Bar, 100 µm. (*H*) Expression levels of (mutant) p53, p53^ER^, and Actb 24 h after vehicle (“−“) or 4OHT (“+”) treatment, based on immunoblotting (see Supplemental Fig. S3D). (*B*,*E*) One-way ANOVA. (*D*,*F*) *t*-test. (ns) Nonsignificant; (*) *P* < 0.05. Data presented were generated from 2D (*A*–*D*,*H*) or 3D (*E*–*G*) cultures of the indicated lung tumor cells.

We next asked whether the transcriptional activity of wild-type p53 in these cells had a functional impact. Restoration of p53 significantly reduced the proliferation of two-dimensional (2D) cultures, and no significant differences were observed between genotypes (Fig. 3A–C; Supplemental Fig. S3A). The anti-proliferative effects of 4OHT were p53^*ER*^-dependent, as 4OHT treatment had no effect on the viability of cells lacking the *p53*^*ER*^ allele (*Kras*^*G12D*/+^;*p53*^*Fx/Fx*^ cells) (Fig. 3D). No senescence or apoptosis was observed upon p53 restoration on 2D cultures (Supplemental Fig. S3B,C). In 3D cultures, p53 restoration also induced comparable anti-proliferative responses in p53-null and mutant cells, but, in these cultures, apoptotic responses were also observed across all genotypes (Fig. 3E–G).

To establish whether the potent tumor-suppressive responses observed were due to wild-type activity rather than mutant p53 depletion (i.e., mutant p53 addiction) (Alexandrova et al. 2015), we examined the impact of wild-type p53 on mutant stability. Wild-type p53 restoration decreased mutant p53 protein levels in most cell lines, indicating that the p53–MDM2 feedback loop was efficiently restored (Fig. 3H; Supplemental Fig. S3D). Nevertheless, following 4OHT treatment, the majority of mutant cells expressed higher levels of mutant than wild-type p53. Hence, p53 restoration induces a robust tumor-suppressive response in both p53-null and mutant lung tumor cells even when the wild-type protein is expressed at relatively low levels (i.e., gene regulated endogenously).

### p53 induces comparable tumor suppression in p53-null and mutant lung tumors in vivo

p53 restoration therapy is an attractive strategy for the treatment of p53-deficient tumors, which already showed efficacy in p53-null lung tumor models (Feldser et al. 2010; Junttila et al. 2010). Our in vitro data now suggest that this therapeutic approach may also be effective in p53 mutant lung tumors, a more clinically relevant p53-deficient tumor cohort. To validate these findings in vivo, independent cohorts of null, R172H, and R270H lung tumor-bearing mice were treated daily for 6 d with vehicle or tamoxifen, which restores p53 functionality.

**Figure 4.**
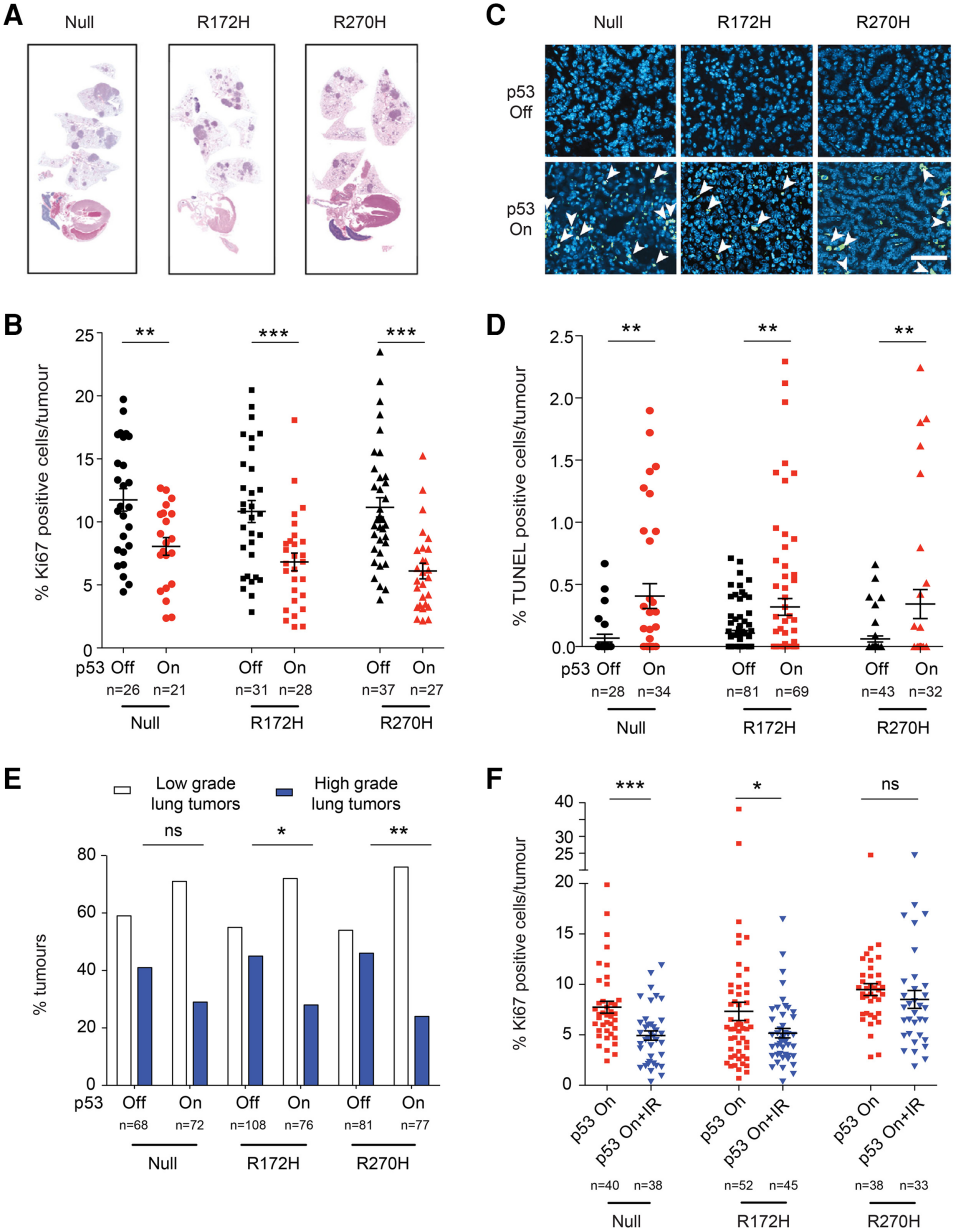
p53 restoration has a comparable therapeutic impact on p53-null and mutant lung tumors in vivo. (*A*) Representative hematoxylin and eosin-stained sections showing lung tumor burden. (*B*) Quantification of Ki67^+^ cells per tumor (immunohistochemistry) in null, R172H, and R270H mice after 6 d of treatment (vehicle: “p53 off”; tamoxifen: “p53 on”). (*C*) Illustrative data showing TUNEL staining (arrowheads) in lung tumor sections. Bar, 60 µm. (*D*) Quantification of TUNEL-positive cells per tumor 24 h after treatment. (*B*,*D*) Circles represent independent tumors, and average value per cohort ±SEM is shown (*t*-test). Three or more mice per cohort were analyzed. (*E*) Quantification of low- and high-grade tumor frequencies in null, R172H, and R270H mice treated for 6 d. A minimum of 68 tumors per cohort (from five or more mice) was analyzed. (*F*) Quantification of Ki67-positive cells per tumor (immunohistochemistry) in null, R172H, and R270H tumors 24 h after treatment with tamoxifen (p53 on) or tamoxifen and irradiation (p53 on + IR). Independent tumor values and average positivity per cohort ±SEM are shown. A minimum of three mice per cohort was used. (*B*,*D*,*F*) *t*-test. (*E*) Fischer's exact test. (ns) Nonsignificant; (*) *P* < 0.05; (**) *P* < 0.01; (***) *P* < 0.001.

Vehicle-treated (p53 off) animals of all genotypes showed similar tumor burden, tumor cell proliferation, and TUNEL positivity as well as comparable tumor grade distribution (Fig. 4A–E; Supplemental Fig. S4A). As seen previously in *Kras*^G12D/+^*;p53*^ER/ER^ animals (Junttila et al. 2010), p53 restoration induced cell cycle arrest and apoptosis and decreased the prevalence of high-grade lung tumors in *Kras*^G12D/+^*;p53*^Fx/ER^ mice (Fig. 4B–E, null). Notably, a similar and significant p53-dependent tumor-suppressive response was observed in R172H and R270H tumors. Indeed, p53-null and p53 mutant tumors exhibited a comparable decrease in proliferation and increase in apoptosis following p53 restoration (Fig. 4B–D). Importantly, all genotypes showed a decrease in the number of high-grade (but not low-grade) lesions following p53 restoration (Fig. 4E), demonstrating that the tumor-suppressive effect of p53 restoration on p53-deficient tumors is tumor grade-specific. In all cases, p53-mediated responses were more prevalent in tumors expressing elevated levels of p19^ARF^ (Supplemental Fig. S4B), indicating that p19^ARF^ induction is the main mediator of p53 activation in these models. Of note, no overall difference in tumor burden could be observed microscopically, likely due to the high proportion of low-grade (and thus irresponsive) tumors present on all genotypes (Fig. 4A; Supplemental Fig. S4A).

Collectively, these data demonstrate that p53 “canonical” tumor suppression (i.e., cell cycle arrest and apoptosis) has a dominant effect over that of mutant p53 in lung tumors in vivo.

### The therapeutic potential of p53 restoration in p53-null and mutant lung tumor cells is enhanced by combined exposure to Nutlin-3 or irradiation

The heterogeneity of p53 activation in lung tumors is a major concern regarding the potential utility of p53 targeted therapy regardless of p53 status. Importantly, p53 activity can be increased by exposure to exogenous p53-activating agents (e.g., Nutlin-3 and irradiation) (Horn and Vousden 2007), but whether such “supra-activation” can be achieved in mutant cells is unclear. The p53-negative regulator MDM2 is often overexpressed in tumors, and its inhibition triggers wild-type p53 activation. MDM2/X inhibitors such as Nutlin-3 can activate wild-type p53 and have shown promising therapeutic effects (Vassilev et al. 2004; Cheok et al. 2011). We thus determined whether Nutlin-3 treatment could enhance the tumor-suppressive potential of wild-type p53 in mutant lung tumor cells. Encouragingly, null, R172H, and R270H cells treated with Nutlin-3 and 4OHT showed a marked decrease in cell viability compared with 4OHT treatment alone (Supplemental Fig. S5A). These data further confirm the therapeutic potential of p53 restoration-based therapy across all p53-deficient genotypes and highlight the need for adequate p53 activation to maximize its efficacy.

We next tested the impact of p53 restoration in combination with irradiation, a common lung cancer therapy. Combined exposure to wild-type p53 and irradiation significantly reduced viability in cell lines from all genotypes (Supplemental Fig. S5B), demonstrating that the activation of wild-type p53 by DNA damage is not impaired by the presence of mutant p53. These results were subsequently validated in lung tumors in vivo. Radiation alone had no significant impact on tumor cell proliferation or apoptosis and did not increase the apoptotic effect of p53 restoration therapy at the time point analyzed (24 h) (data not shown). Of note, short-term (24 h) (Fig. 4F) and long-term (6 d) (Fig. 4B) p53 restoration had similar anti-proliferative and anti-apoptotic (data not shown) effects, indicating that the tumor-suppressive impact of wild-type p53 is sustained over time across all genotypes. Importantly, combined p53 restoration and irradiation treatment resulted in an enhanced cytostatic effect relative to p53 restoration (Fig. 4F), demonstrating that the in vivo impact of p53 targeted therapy can be improved through exogenous activating signals. However, the enhanced efficacy of this combined treatment was less marked in R270H tumors than in p53-null and R172H lesions, suggesting that p53-deficient lung tumors may exhibit genotype-specific therapeutic vulnerabilities.

### R172H and R270H p53 mutants exhibit transcriptional GOFs in lung tumor cells

Our data show that p53 mutations are unlikely to be a major obstacle for the efficacy of p53 restoration-based lung cancer therapy. Nevertheless, our transcriptional analyses uncovered p53 mutation type-specific signatures in lung tumor cells (Fig. 1C,D), suggesting that these mutants may exhibit distinct susceptibilities in other contexts. Since p53 genotype-specific signatures may have therapeutic implications, we carried out an unbiased comparative analysis of all p53 genotypes under normal conditions (p53 off) to identify putative mutant GOFs.

**Figure 5.**
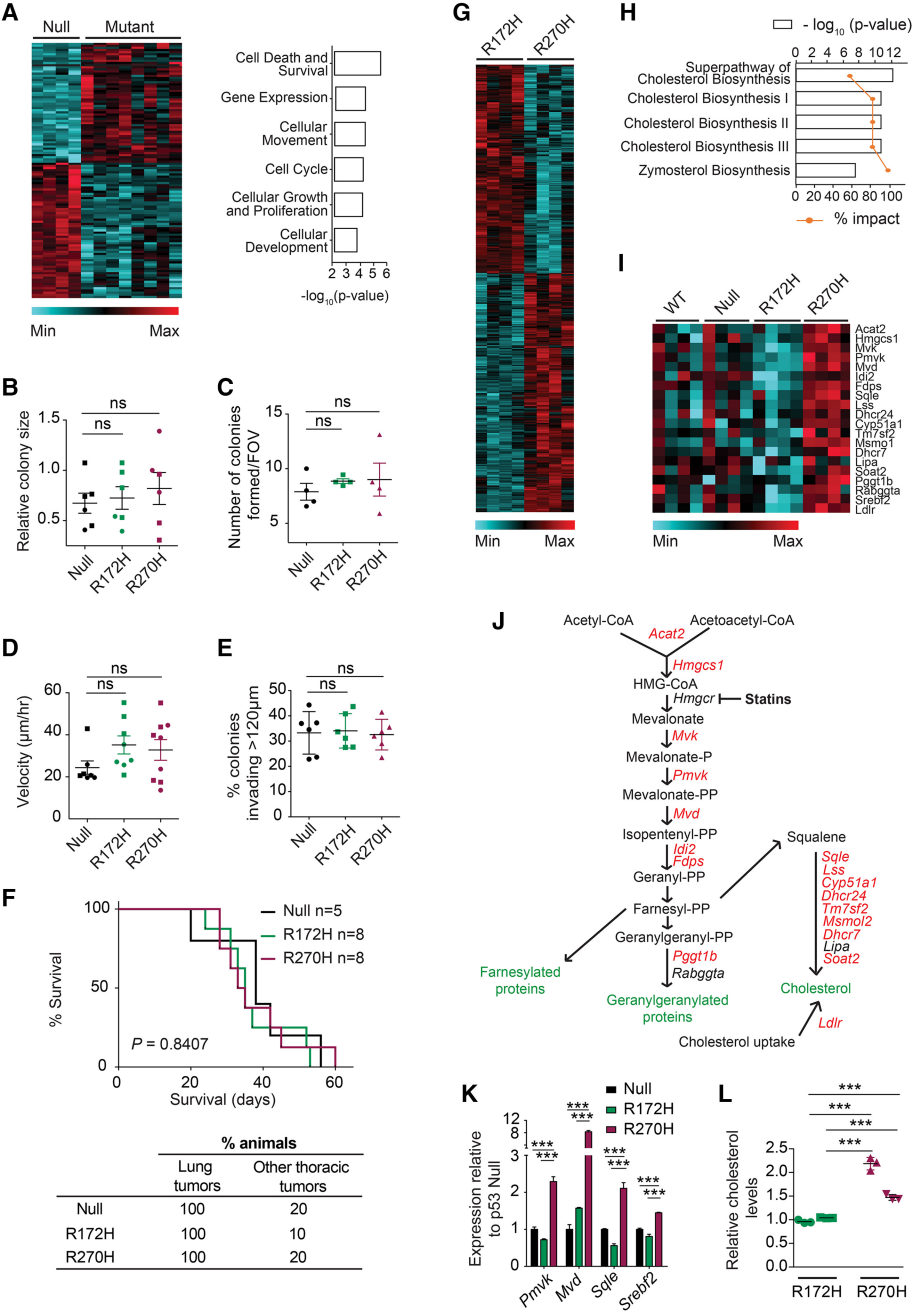
R172H and R270H mutants exhibit both common and mutant type-specific transcriptional GOFs. (*A*, *left*) Heat map depicting genes differentially expressed between p53-null and p53 mutant (R172H + R270H) cell lines, based on microarray analysis. (*Right*) Molecular functions most significantly altered between the genotypes shown (Ingenuity Pathway Analysis [IPA]). (*B*) Average colony sizes of independent p53 mutant and p53-null cell lines (symbols) grown in triplicate in 3D for 72 h, relative to internal control, are shown ±SEM. (*C*) The number of colonies per field of view formed by p53 mutant and p53-null cell lines (symbols) grown in 3D ±SEM. (*D*) Migration potential of the indicated genotypes (scratch assay). Mean velocity per genotype ±SEM is shown. Independent tumor cell lines from both *p53*^*Fx*^-derived (squares) and *p53*^*ER*^-derived (circles) models are shown. (*E*) Representative data (*n* = 2 per genotype) show the percentage of p53-null and p53 mutant colonies that invaded >120 µm through Matrigel. Replicates for one cell line per genotype ±SD are shown. (*F*) Survival (*top*) and tumor frequency (*bottom*) of recipient mice transplanted (intravenously) with cell lines of the indicated genotype. Log-rank (Mantel-Cox) test. (*G*) Heat map depicting genes differentially expressed between R172H and R270H lung tumor cells. (*H*) The top canonical pathways significantly altered between R172H and R270H cells (IPA). (*I*) The expression of mevalonate (MVA) pathway genes in null, R172H, and R270H cells is displayed (heat map). Null cells are shown in the absence (“null”) and presence (“wild-type”) of 4OHT treatment (8 h). (*J*) Schematic representation of the MVA pathway, with genes significantly deregulated between R172H and R270H cells highlighted in red. *t-*test, *P* < 0.05. (*K*) The expression of the indicated genes in null, R172H, and R270H cells (3D) was analyzed by TaqMan and normalized to *Gapdh*. Representative data (*n* = 3 runs) show one cell line per genotype ±SD of triplicates. (*L*) Intracellular cholesterol levels of R172H and R270H cells relative to average R172H values and normalized to protein levels. Representative data show two cell lines per genotype (represented by different symbols) *n* = 4 analyzed per genotype, and ±SD of triplicates is indicated. (*B*–*E*,*K*) One-way ANOVA. (*L*) *t*-test. (ns) Nonsignificant; (***) *P* < 0.001.

Multiple genes (*n* = 465) were differentially expressed in mutant (R172H + R270H) lung tumor cells relative to null, indicative of mutation type-independent mutant p53 (i.e., “universal”) transcriptional GOFs. Genes involved in cell death and survival, cellular movement, and cell cycle topped the list of molecular functions significantly altered (Fig. 5A), suggesting that p53 mutations may affect the proliferative and invasive capacity of lung cancer cells, as described in other tumor types (Freed-Pastor et al. 2012; Weissmueller et al. 2014). However, no significant differences in colony size, number, or invasiveness (Fig. 5B–E; Supplemental Fig. S6A–C) or tumor cell metastatic potential in vivo (Fig. 5F; Supplemental Fig. S6B,C) could be detected between null, R172H, and R270H cells using both *p53*^*ER*^- and *p53*^*Fx*^-derived models. These data show that the GOF transcriptional signatures of p53 mutant cells do not necessarily result in measurable GOF cellular phenotypes and that p53-null, p53 contact, and p53 conformational mutant lung tumor cells have comparable proliferative capacity and invasive potential.

Unexpectedly, our comparative analysis revealed significant transcriptional distinctions between R172H and R270H cells, with 972 genes being differentially expressed. Lipid metabolism—and the mevalonate (MVA) pathway in particular—was the most differentially regulated pathway (Fig. 5G–I). The MVA pathway regulates multiple cellular processes through the production of a wide range of molecules, such as cholesterol and farnesylation and geranylgeranylation intermediates (Mullen et al. 2016). Up-regulation of MVA pathway genes was associated previously with mutant p53 activity in breast cancer cells (Freed-Pastor et al. 2012). Our data indicate that this GOF may not be universal but rather mutation type-specific. Indeed, the majority of MVA pathway genes was up-regulated in R270H mutant cells relative to R172H (Fig. 5I–K). R270H MVA pathway gene expression was also enhanced relative to p53-null and “wild-type” cells (null + 4OHT) (Fig. 5I). Wild-type p53 restoration did not restore normal MVA pathway gene expression in R270H cells (Supplemental Fig. S6D, null p53 on vs. R270H), demonstrating the dominance of this R270H mutant phenotype.

### p53 R270H lung tumor cells exhibit increased dependency on MVA pathway gene expression relative to R172H cells

Given that cholesterol is a major product of the MVA pathway (Mullen et al. 2016), we assessed cholesterol levels in our cultures and found a significant increase in R270H tumor cells (Fig. 5L), which functionally validated the transcriptional GOF observed. Under physiological conditions, the MVA pathway is activated by low intracellular sterol concentrations or increased demand for cholesterol (Mullen et al. 2016). Our data suggest that this “feedback loop” may be deregulated in R270H cells, but the relevance of this transcriptional phenotype was unclear.

**Figure 6.**
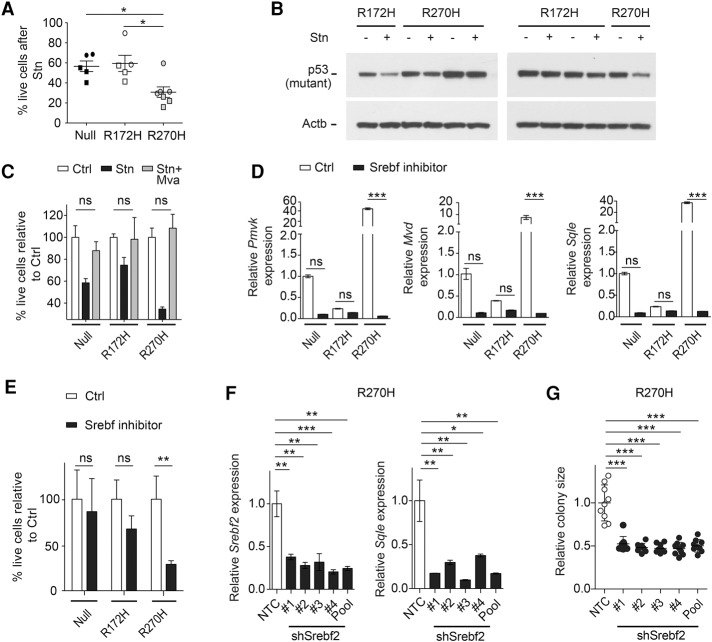
Enhanced sensitivity of p53 R270H lung tumor 3D cultures to MVA pathway inhibition. (*A*) Representative data (*n* = 2 runs) show the percentage of viable cells following 72 h of statin (Stn) treatment, relative to vehicle. Symbols represent independent tumor cell lines from both *p53*^*Fx*^-derived (squares) and *p53*^*ER*^-derived (circles) models. SEM per genotype is shown. (*B*) Expression of mutant p53 and Actb (immunoblotting) in three R172H and R270H cell lines 24 h after Stn (+) or vehicle (−) treatment. (*C*) Viability of cells following 72 h of Stn or Stn + MVA treatment, relative to vehicle (Ctrl). Representative data for one cell line per genotype are shown. *n* = 3 tested; ±SD of triplicates. (*D*) Expression of the indicated genes (TaqMan) in p53-null, R172H, and R270H cells in the presence (Srebf inhibitor) or absence (Ctrl) of the Srebf inhibitor fatostatin (72 h). (*E*) Cell viability 72 h after treatment of the indicated cells with fatostatin or Ctrl. (*D*,*E*) Representative data (of *n* = 3 runs) show one cell line per genotype ±SD of triplicates. (*F*) Expression of the indicated genes (TaqMan) in R270H cells following shRNA knockdown of Srebf2 with four different constructs (#1–#4) or all four constructs (pool). (*G*) Average colony size per field of view of R270H cells following Srebf2 knockdown. (*F*,*G*) Representative expression data (of *n* = 2 runs) shows one cell line per genotype ±SD of triplicates, relative to nontargeting control (NTC). (*A*) One-way ANOVA. (*C*,*F*,*G*) *t*-test. (*D*,*E*) Two-way ANOVA. (ns) Nonsignificant; (*) *P* < 0.05; (**) *P* < 0.01; (***) *P* < 0.001. All data were obtained from 3D cultures of the indicated genotypes.

We therefore asked whether R270H cells had an increased dependency on MVA pathway function compared with other p53-deficient cells. The MVA pathway can be efficiently down-regulated by statins (Fig. 5J), a group of drugs widely used in the clinic to lower de novo cholesterol biosynthesis and serum low-density lipoprotein cholesterol levels through the specific inhibition of HMG-CoA reductase (HMGCR) (Istvan and Deisenhofer 2001; Cholesterol Treatment Trialists Collaboration 2010). To compare their sensitivity to MVA pathway inhibition, p53-null, R172H, and R270H 3D lung cell cultures (combining *p53*^*Fx*^ and *p53*^*ER*^ models) were treated with statin (simvastatin [Stn]) or vehicle for 72 h. Statin treatment resulted in decreased colony growth across all genotypes. Nevertheless, this effect was more significant in R270H cells (Fig. 6A). The enhanced sensitivity of R270H cells to statin was not due to preferential degradation of this mutant protein by the treatment (Fig. 6B), as described previously for conformational p53 mutants in a different context (Parrales et al. 2016).

The reduced growth of lung tumor cultures following statin treatment was fully rescued by MVA administration, demonstrating that the effects of statin treatment were due to MVA pathway inhibition (Fig. 6C). MVA pathway gene expression is regulated by the transcription factor Srebf2 (also known as Srebp2), which was also up-regulated in R270H cells (Fig. 5I,K). To determine whether Srebf2 activity was essential to the MVA pathway phenotype of R270H cells, its cleavage and subsequent activation were inhibited by the administration of the Srebf inhibitor fatostatin (Kamisuki et al. 2009). Fatostatin treatment inhibited the expression of MVA pathway genes in all genotypes (Fig. 6D). However, similarly to statin, fatostatin treatment was significantly more deleterious to R270H cultures than to R172H or null (Fig. 6E). Since upon fatostatin treatment all genotypes expressed comparable levels of MVA pathway genes, these data demonstrate that R270H lung tumor cells are significantly more dependent on this pathway than other p53-deficient genotypes. Srebf2 knockdown recapitulated the effects of fatostatin on R270H cells, confirming the dependence of these cells on Srebf2 for MVA pathway gene expression and colony growth (Fig. 6F,G; Supplemental Fig. S6E). These data suggest that abnormal regulation of Srebf2 is an important transcriptional GOF of p53^R270H^ in lung tumor cells.

### Statin treatment induces a robust therapeutic response in spontaneous p53^R270H^ mutant (but not p53^R172H^) Kras^G12D^-driven lung tumors

**Figure 7.**
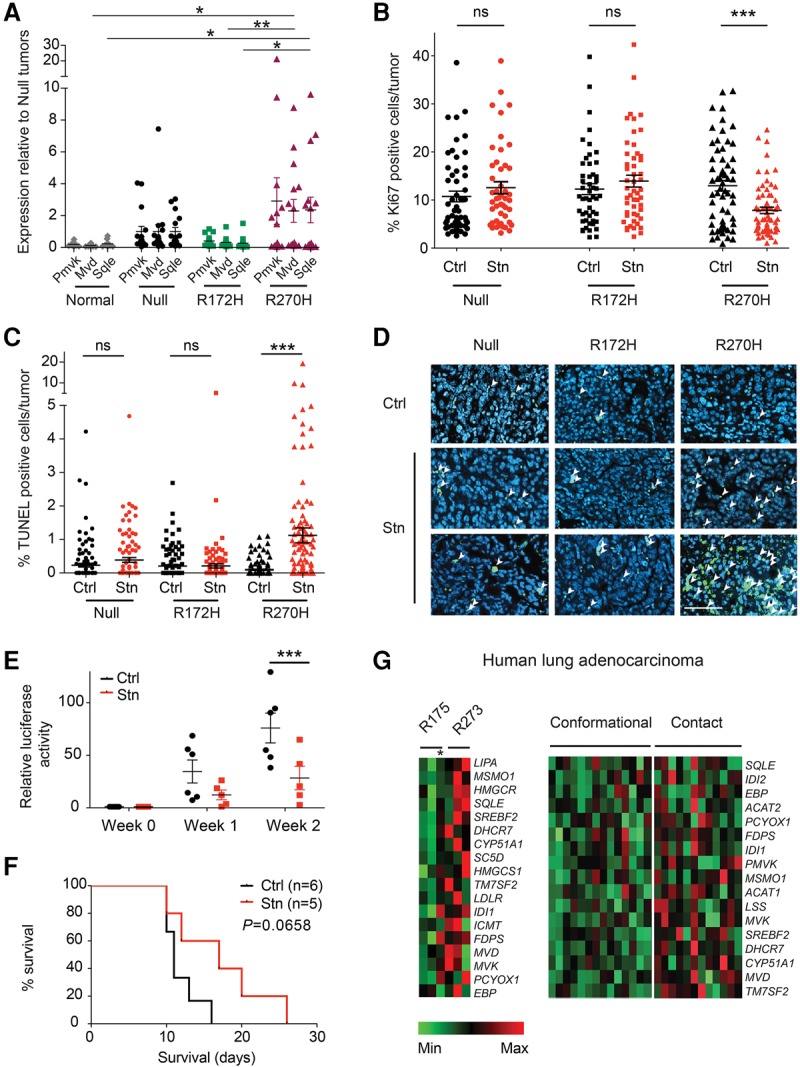
Increased MVA gene expression and sensitivity to statin treatment in p53 contact mutant lung tumors. (*A*) Expression of *Pmvk*, *Mvd*, and *Sqle* (TaqMan) in null, R172H, and R270H lung tumors (Fx model) normalized to 18S expression. Expression is shown relative to the average of null tumors ±SEM per genotype. *n* = 10 normal lungs; *n* = 15 tumors per genotype; *n* ≥ 4 mice per cohort. (*B*) The percentage of Ki67-positive cells per tumor in vehicle-treated (Ctrl) or Stn-treated (6 d of treatment) null, R172H, and R270H mice. A minimum of 47 tumors was analyzed. *n* = 5 mice per cohort. The average positivity per cohort ±SEM is shown. (*C*) The percentage of TUNEL-positive cells per tumor in Ctrl or Stn-treated mice (6 d of treatment). A minimum of 93 tumors (*n* = 5 mice) was analyzed per cohort. (*A*–*C*) Symbols depict independent normal tissues per tumor samples. (*D*) Representative TUNEL staining (arrowheads) in lung tumor sections from Ctrl or Stn-treated mice. Bar, 50 µm. Lung tumor burden (luciferase activity; *E*) and survival (*F*) of recipient mice transplanted (intravenously) with R270H lung tumor cells and treated with vehicle or Stn. *n* ≥ 5 mice per cohort. Daily treatments were started (week 0) 14 d after transplantation. Log-rank (Mantel-Cox) test. (*G*) Heat maps depicting expression of MVA pathway genes significantly up-regulated in human p53 contact mutant lung adenocarcinomas relative to conformational mutant tumors. Samples with a R175 or R273 mutation are shown independently (*left*) or combined with other known conformational and contact mutations (*right*) (see Supplemental Table S3). An asterisk denotes a tumor with a R175H and a C277F (contact) mutation. (*A*–*C*,*E*) *t-*test. (*) *P* < 0.05; (**) *P* < 0.01; (***) *P* < 0.001.

To test the relevance of this p53 R270H-specific GOF in vivo, we generated lung tumors in *Kras*^G12D/+^*;p53*^Fx/*Fx*^, *Kras*^G12D/+^*;p53*^*R172H/Fx*^, and *Kras*^G12D/+^*;p53*^*R270H/Fx*^ mice. For consistency, these tumors are again referred to here as null, R172H, R270H, respectively (Fig. 7A–D). Similarly to in vitro data*,* R270H lung tumors expressed significantly higher levels of the MVA pathway genes *Mvd* and *Sqle* than normal lung and R172H lesions (Fig. 7A). To test the sensitivity of these tumors to MVA pathway inhibition, lung tumor-bearing mice were treated with vehicle (Ctrl) or Stn (statin) for 6 d. In vivo, statin treatment had no significant anti-proliferative or apoptotic effect on p53-null and R172H lung tumors (Fig. 7B,C). In contrast, R270H tumors showed a striking response to statin treatment, which impacted both tumor cell proliferation (R270H mean values: 13% Ctrl; 7% Stn) and survival (<0.1% vs. >1% average TUNEL^+^ cells in vehicle [Ctrl] vs. Stn-treated samples, respectively). Notably, the dramatic cytotoxic effect of statin treatment on p53 R270H lung tumors was characterized by an increase in both the number of TUNEL-positive tumors and TUNEL positivity per tumor (Fig. 7C,D; data not shown). No effect of statin on tumor grade distribution was observed, suggesting that this treatment is similarly effective in low- and high-grade lung tumors (Supplemental Fig. S6F).

Continuous, long-term statin treatment of an R270H orthotopic lung tumor model significantly reduced tumor burden and improved host survival, demonstrating the therapeutic potential of this statin on lung tumors harboring this particular p53 mutation (Fig. 7E,F). Finally, we asked whether the p53 mutation specificity of MVA pathway deregulation was also observed in human tumors. For that purpose, RNA sequencing (RNA-seq) data from The Cancer Genome Atlas (TCGA) lung adenocarcinomas with R175, R273, or other known conformational and contact p53 mutations (Supplemental Table S3) were analyzed. As shown in Figure 7G, the expression of MVA pathway genes was significantly enhanced in R273 relative to R175 mutant tumors but also in other lesions with p53 contact mutations relative to conformational mutant tumors. Hence, human lung cancer exhibits a p53 mutation type-specific GOF similar to that seen in murine tumors and thus, potentially, a comparable therapeutic sensitivity.

Our comprehensive profiling of DN and GOF effects of distinct p53 mutants enabled the identification of mutant p53 lung tumor vulnerabilities that can be potentially exploited therapeutically. Importantly, our study also demonstrated that some of these liabilities are likely to be mutation type-dependent.

## Discussion

Lung cancer is the leading cause of cancer-related death worldwide, reflecting its high incidence and the limited efficacy of available therapies. The identification of therapeutic vulnerabilities in p53 mutant lung tumors provides important targeting opportunities for a significant proportion of lung adenocarcinomas. Given the accumulation of p53 mutants in tumors, their oncogenic impact can go beyond loss of wild-type function. Indeed, mutant p53 DN and GOF phenotypes have been described (Freed-Pastor and Prives 2012; Bieging et al. 2014), but whether these are universal phenotypes or tumor and/or mutation-specific traits remained unclear. Our study provides the first evidence of DN and GOF effects of distinct p53 mutants in lung tumors and identifies therapeutic vulnerabilities associated with distinct p53 alterations. We show that p53 binding and conformational mutants exhibit both common and distinct therapeutic vulnerabilities relative to p53-null tumors and even between each other.

Our data argue against a major DN effect of mutant p53 in lung tumor cells. Indeed, while we found evidence of transcriptional DN activity for both mutants, multiple key p53 targets were efficiently regulated by wild-type p53 in mutant cells. It has been proposed that p53 mutants interfere with wild-type function through direct binding to the wild-type protein, with the resulting heterotetramers being transcriptionally impaired (Milner and Medcalf 1991; Chan et al. 2004). Interestingly, analysis of ChIP data sets (http://chip-atlas.org; Fischer 2017) revealed that the large majority (64%–82%) of genes similarly regulated by wild-type p53 across all genotypes is direct p53 targets. Since these targets were induced in cells expressing higher levels of mutant than wild-type protein, these data suggest that p53 mutant:wild-type heterotetramers are likely to retain some wild-type activity in lung tumor cells. Alternatively, these transcriptional profiles may be explained by a bias of the wild-type protein toward homotetramer formation in these cells. In contrast, ChIP data analysis revealed that only 41%–55% of genes associated with DN mutant signatures are direct p53 targets, suggesting that mutant p53 may exert some of these effects by interfering with the activity of transcription factors induced by wild-type p53.

Importantly, the transcriptional activity retained by wild-type p53 in mutant p53 lung tumors was sufficient to induce anti-proliferative and apoptotic responses similar to those observed in p53-null samples, potentially explaining the selective pressure for the loss of the wild-type allele in mutant p53 lung tumors in humans. This dominant effect of the wild-type p53 protein has important therapeutic implications. Indeed, since the DN activity of mutant p53 was unable to prevent wild-type p53 tumor suppression in lung tumors in vivo, our data show that wild-type p53 restoration and activation strategies should be equally effective on p53-null and mutant tumors independently of the p53 mutation present.

It is possible that the dominant effect of wild-type p53 in p53 mutant cells is lung tumor-specific, as wild-type p53 restoration was shown previously to induce a more potent effect in p53-null lymphomas and angiosarcomas than in mutant lesions (Wang et al. 2011). However, in that study, p53 restoration involved the use of a hypomorphic wild-type p53, potentially explaining the discrepancy with our findings.

Murine studies suggest that p53 targeted therapy may be of benefit only to patients with advanced lung tumors, as p53 is adequately activated only in those lesions (Feldser et al. 2010; Junttila et al. 2010). Nevertheless, this approach has the potential to benefit a large patient population, as most lung tumors are diagnosed at advanced stages. Furthermore, we observed similar anti-proliferative and apoptotic responses after 24 h and 6 d of treatment, suggesting that p53 restoration can provide long-term protection against high-grade tumor cells. Importantly, we show that the in vivo efficacy of this therapy can be improved through exogenous p53 activation (e.g., irradiation). Since lung cancer therapy relies strongly on DNA damage-inducing agents (Reck et al. 2013), the therapeutic impact of p53 restoration is likely to be enhanced by standard therapy. Conversely, our data imply that functional nonactive wild-type p53 may be retained in mutant p53 low-grade tumors, where its activity can be potentially engaged therapeutically.

Surprisingly, we were unable to identify a common (R172H + R270H) functional GOF in lung tumor cells or evidence of enhanced invasiveness/metastatic potential of p53 mutant cells relative to p53-null. While this is consistent with the comparable lung tumor development kinetics reported for p53-null and mutant mice (Jackson et al. 2005), it is in striking contrast to data from PDAC (Weissmueller et al. 2014) and breast cancer (Freed-Pastor et al. 2012) models. Interestingly, mutant p53 lung tumor cells showed discrepancies with these models at both transcriptional (unlike in PDAC, Pdgfrb was not overexpressed in mutant lung tumor cells) and functional (unlike in breast models, MVA pathway gene up-regulation did not increase lung tumor cell invasiveness) levels. These data demonstrate that mutant p53 GOF phenotypes are tissue-specific and imply that the vulnerabilities of mutant p53 tumors are likely to be tissue/context-dependent.

Unexpectedly, we found that p53 conformational and contact mutant murine and human lung tumors exhibit distinct transcriptional signatures. In particular, R270H mutant tumors displayed enhanced MVA pathway gene expression, and R270H murine cell lines showed a high dependence on this transcriptional signature. Indeed, MVA pathway inhibition through simvastatin treatment induced robust cell cycle arrest and apoptotic responses in R270H lung tumors but had no significant impact on p53-null or R172H lung tumors.

It is unclear how p53 mutants exert mutation-specific transcriptional phenotypes, but these effects likely involve differential mutant binding to wild-type p53 (DN effects) or other transcriptional factors (DN and GOF effects) (Freed-Pastor and Prives 2012). Alternatively, the mutants may bind the same factors but exhibit differential cooperativity and thus distinct promoter affinities (Schlereth et al. 2013).

This study shows that the analysis of lung tumor subset-specific transcriptional signatures can lead to the identification of tumor vulnerabilities to agents already available in the clinic, as shown here for simvastatin. Importantly, TCGA data suggest that the R270H-dependent MVA pathway GOF may be shared by other DNA contact mutants in human lung tumors. Notably, while the effects of statins on cancer remain controversial (Mullen et al. 2016), there are reports of improved survival among statin-treated lung cancer patients (Cardwell et al. 2015; Lin et al. 2016). Based on our findings, it would be important to determine whether mutant p53 status is associated with that benefit. Moreover, as seen in our models, MVA gene expression profiling may help identify lung cancer patients that could profit from statin repurposing for cancer therapy.

## Materials and methods

### Mice, adenoviral infection, and treatments

This research was regulated under the Animals (Scientific Procedures) Act 1986 Amendment Regulations 2012 following ethical review by the University of Cambridge Animal Welfare and Ethical Review Body (AWERB). For the generation of endogenous lung tumor models, mixed background (C57Bl/6/129/Sv) *Kras*^*LSL-G12D*/+^;*p53*^*LSL-R270H/ER*^, *Kras*^*LSL-G12D*/+^;*p53*^*LSL-R172H/ER*^, *Kras*^*LSL-G12D*/+^;*p53*^*Fx/ER*^, *Kras*^*LSL-G12D*/+^;*p53*^*LSL-R270H/Fx*^, *Kras*^*LSL-G12D*/+^;*p53*^*LSL-R172H/Fx*^, and *Kras*^*LSL-G12D*/+^;*p53*^*Fx/Fx*^ mice were generated (Jonkers et al. 2001; Christophorou et al. 2005; Jackson et al. 2005). Lung tumors were induced by intranasal administration of Cre-expressing adenovirus, as described previously (5 × 10^6^ plaque-forming units per mouse; University of Iowa Vector Core) (Junttila et al. 2010; Kerr et al. 2016). For p53 restoration studies, mice were treated at 15–17 wk after Cre administration by intraperitoneal injection with 1 mg of tamoxifen (Sigma) per mouse once per day (Junttila et al. 2010) or vehicle (peanut oil) for 1 or 6 d, as indicated. For combined treatments, mice were irradiated with 4 Gy, (cesium source) 2 h after a single tamoxifen/vehicle treatment. Stn (200 mg/kg; EP, S0650000) or vehicle (1% carboxymethylcellulose in water) were administered 15 wk after Cre, once daily for 6 d by oral gavage. Transplant studies were carried out as described previously (Kerr et al. 2016) using one cell line per genotype. Prior to transplantation, lung tumor cells were transduced with MSCV-luciferase-hygromycin retrovirus and selected (350 µg/mL hygromycin B). Recipient mice received either 1 × 10^5^ cells (metastatic potential cohorts) or 1 × 10^6^ cells (Stn/Ctrl cohorts) by intravenous injection. All plotted mice developed lung lesions; additional thoracic tumors were observed in some mice. Animals were imaged weekly using an IVIS Spectrum Xenogen machine (Caliper Life Sciences) as described previously (Kerr et al. 2016). Stn or control vehicle treatment of transplanted models was carried out once daily starting 2 wk after transplantation and until endpoint. In all in vivo treatment groups, lungs were collected 24 h after the last treatment, and a minimum of three mice per cohort was used (exact values are indicated).

### Immunohistochemistry, immunofluorescence, and tumor grading

Histological analysis was carried out on formalin-fixed, paraffin-embedded (5-µm) lung tissue sections. Tumor grading was performed on hematoxylin and eosin (H&E)-stained sections according to Nikitin et al. (2004) and Junttila et al. (2010) (low grade: adenomas and grade 1 and 2 adenocarcinoma; high grade: grade 3 and 4 adenocarcinoma). Tumor burden was measured from H&E sections containing a minimum of four lung lobes per section (one section per animal analyzed). The total area of the section covered by tumors was measured and then calculated as a percentage of the total lung area on the section using ImageJ software. The following primary antibodies were used: Ki67: immunofluorescence, RM-9106 (1:120; Thermo Scientific), immunohistochemistry, IHC-00375 (1:200; Bethyl Laboratories); cleaved caspase-3: 9664S (1:200; Cell Signaling); and p19^ARF^: MAB2417 (1:500; Novus). The following corresponding secondary antibodies were used: A11008 (1:200) and A21471 (1:200), both from Thermo Scientific. TUNEL positivity was assessed using ApopTag kit (Millipore), and tumors were considered positive if five or more positive nuclei were found per field of view. The minimum cohort sizes used are indicated.

### Generation of lung tumor cell lines

Lung tumor cell lines were generated from independent tumors, and a minimum of two mice were used per genotype. Tumors were collected in Hank's balanced salt solution (HBSS), mashed, and digested with 4 mg/mL collagenase/dispase (Roche) for 2.5 h at 37°C. Cells were treated with HBSS containing 10% FCS and DNase I (Invitrogen), filtered (70 µm), and plated in DMEM/F-12 medium (Life Technologies) with 10% FCS and 4 mM L-Glutamine. After 1.5 h, the medium (still containing floating cells) was replated, and tumor cell-enriched (medium: slower adherence) and TAF-enriched (fast adherence) cultures were maintained separately. Epithelial and fibroblast content was assessed by EpCAM (Biolegend, 118213) and Pdgfra (eBioscience,17-1401-81) FACS analysis using a LSRII (BD) flow cytometer and FlowJo software (TreeStar). All tumor cell lines used were <8% Pdgfra^+^. All cell lines used were propagated in DMEM/F12 medium, tested negative for mycoplasma, and were typically used between passages 10 and 20 after isolation.

### 3D cell cultures

To generate 3D cultures, cells were seeded in a 3:2 Matrigel (Corning, 354234):DMEM/F12 mix and allowed to set at 37°C before additional medium was added. For cell harvesting, Matrigel was dissociated by incubation with Corning cell recovery solution (354253) for 45 min at 4°C, and cell pellets were collected (RNA/protein analysis) or cells were trypsinized (cell counts/FACS analysis). To assess colony formation, 5000 cells were seeded in Matrigel as above. After 72 h, three to five images were taken for each well at 20× magnification, and colony size was measured using Image J software. For immunofluorescence, 3D cultures were fixed in 2% PFA for 1 h followed by 1% glutaldehyde for 1 h and then were blocked in 0.15% PBS and 3% Triton-X serum for 30 min. Antibodies were used at the indicated concentrations with overnight incubations. For Srebf2 knockdown, cells were transiently transfected with 10 µg of GFP^+^ shRNA plasmids (TG510683A-D) or control nontargeting sequence (TR30013) from Origene. Forty-eight hours after transfection, cells were sorted for GFP positivity, and 50,000 GFP^+^ cells were seeded in Matrigel/DMEM-F12 medium. Colony size was measured 72 h after seeding as above, and cells were collected for RNA extraction.

### In vitro assays and treatments (2D and 3D cultures)

For growth curve generation, 2D cultures were treated in triplicate with 100 nM 4OHT (Sigma, H7904) or Ctrl (ethanol), and cell viability was analyzed by Trypan blue exclusion at the indicated time points after treatment start. Where indicated, cells were administered 10 µM Nutlin3 (VWR)/vehicle (DMSO) or irradiated once with 4 Gy (Xstrahl X-ray irradiator). Combination treatments were carried out as follows: For irradiation, 4OHT/vehicle was provided 2 h before irradiation, and cell viability was assessed 48 h after irradiation; for Nutlin3, 4OHT/vehicle and Nutlin3/vehicle were administered simultaneously, and the medium and drugs were refreshed daily. Cell viability was assessed 24, 48, and 72 h after the beginning of the treatment. Senescence was assessed 72 h after 4OHT/Ctrl treatment (2D cultures) using a β-galactosidase staining kit (Cell Signaling, 9860). p53 wild-type lung cancer cells (A549 and H460) treated with 2 µM palbociclib (PD-0332991) for 72 h were used as a positive control. Images were obtained from five random fields of view for each cell line in triplicate. For BrdU/PI FACS analysis, 2D and 3D cultures were treated with 100 nM 4OHT/Ctrl for 72 h and processed as described previously (Kerr et al. 2016). Cell death following 72 h of 4OHT/Ctrl treatment was measured in both 2D and 3D cultures by the ethidium homodimer/calcein AM kit (Molecular Probes, MP03224). Stn was activated prior to use as described previously (Freed-Pastor et al. 2012). Stn (1 µM; Sigma), 10 µM fatostatin (Sigma) in DMSO, 0.3 mM MVA (Sigma) in DMSO, and the corresponding vehicles were refreshed daily, and viability was assessed by Trypan blue exclusion (72 h). For scratch assays, cells were cultured for 48 h before each well was scratched twice. Scratch closure was monitored by Leica live-cell microscopy with images acquired hourly for 24 h. Velocity was calculated from three measurements per scratch per cell line, and the average value was plotted. Invasive growth assays were performed as reported (Zhou et al. 2014) without addition of EGF to the medium. Intracellular cholesterol levels (2D cultures) were measured using the Amplex Red cholesterol kit (Molecular Probes, A12216), and values were normalized to total protein content. A minimum of two independent cell lines per genotype was used in all assays (typically three or four, as indicated), except for fatostatin and shRNA experiments (one cell line per genotype). Experimental triplicates were used for all assays.

### Microarray analysis, TaqMan validation, and TCGA analysis

Microarray analysis was performed on four independent murine lung tumor cell lines per genotype cultured in 3D Matrigel. Cells were cultured for 48 h and then treated with either ethanol (vehicle) or 100 nM 4OHT for 2 or 8 h. Cells were harvested following dissociation of Matrigel, and RNA was extracted (RNeasy, Qiagen). Microarray analysis was carried out using Illumina MouseWG-6 version 2.0 Expression BeadChip (Department of Pathology, Cambridge University). Arrays were scanned using standard Illumina protocols, and data were analyzed using R (http://www.R-project.org) and Bioconductor (Gentleman et al. 2004). Spatial artifacts were removed using BASH (Cairns et al. 2008) and HULK algorithms from the Beadarray package (Dunning et al. 2007). Data were log_2_-transformed and quantile-normalized. Differentially expressed genes were defined as an average fold change between groups >1.4 and SD < 0.5 within each cohort to account for intragenotype variation. Ingenuity Pathway Analysis (IPA) software (http://www.ingenuity.com) was used for pathway analysis of differentially expressed genes, and statistical significance (*P* < 0.05) of canonical pathways was determined by Fisher's exact test. Heat maps were generated using Multiple Experiment Viewer (MeV). Gene expression changes were validated by quantitative PCR using Life Technologies probes. Samples were run in triplicate, and the data were normalized to 18S or GAPDH expression. p53 “canonical” targets were defined based on functional/expression studies (gene set enrichment analysis) (Okamoto and Beach 1994; Lehar et al. 1996; Utrera et al. 1998; Bieging et al. 2014) or ChIP data (http://chip-atlas.org); Fischer 2017). Lung adenocarcinoma patient TCGA (The Cancer Genome Atlas Research Network 2014) data were downloaded from cBioportal, and cases with *TP53* mutations were divided into conformational, contact, nonmissense, or unclassified based on the *TP53* mutation. Samples with mutations classified as conformational or contact by Cho et al. (1994), Joerger et al. (2006), and Joerger and Fersht (2007) were analyzed for expression of MVA pathway genes (RNA-seq V2 RSEM). Significance was calculated based on the Significance Analysis for Microarrays (SAM) platform (Tusher et al. 2001) within MeV.

### Western blotting

Cells lysates were loaded onto 4%–12% Bis-Tris gels, separated by electrophoresis, and transferred onto a methanol-activated PVDF (Immobilon) membrane. Primary antibodies against p53 (1:1000; Vector Laboratories, CM5, VP-P956) and Actb (1:10,000; Sigma, A5441) and their corresponding secondary antibodies (HRP-linked-anti-rabbit [1:2000; Cell Signaling, 7074S] or anti-mouse [1:3000; Invitrogen, 626520]) were used.

### Genotyping and TaqMan probes

Mice and lung cell lines were genotyped as described previously for *Kras*^*G12D*^ (https://jacks-lab.mit.edu/protocols) and *p53*^*Fx*^ (Jonkers et al. 2001). The p53ER PCR primers used were (1) 5′-CCTCCAGCCTAGAGCCTTCCAAGC-3′, (2) 5′-GGTGAGATTTCATTGTAGGTGCC-3′, and (3) 5′-GCACACAAACTCTTCACCCTGC-3′. The wild-type band used was 430 base pairs (bp), and the KI band used was 700 bp. Mutant p53 sequencing was carried out using the following primers: 172: ([1] AGGTGTGTTGGCCATCTCTG and [2] CTCAGGAGGGTGAGGCAAAC) and 270 ([1] TTTCTGTTCCACGAGTCCCG and [2] AAAAGACCTGGCAACCTGCT). The following probes were used for TaqMan analysis: *Cdkn1a* (Mm00432448_m1), *Mdm2* (Mm01233136_m1), *Bbc3* (Mm00519268_m1), *Mvd* (Mm00507014_m1), *Pmvk* (Mm01212763_m1), *Sqle* (Mm00436772_m1), *Srebf2* (Mm01306292_m1), *Pilra* (Mm04211819_m1), *Nr2f2* (Mm00772789_m1), *Slc2a9* (Mm00455122_m1), *Phlda3* (Mm00449846_m1), *Sesn2* (Mm00460679_m1), *Bax* (Mm00432051_m1), *Gapdh* (Mm99999915_g1), and Euk 18S rRNA (4352930E), all from Applied Biosystems.

### Statistical analyses

Statistical analyses were performed using Prism 5.0 software (Graphpad), with *P* < 0.05 considered statistically significant. *P-*values for unpaired comparisons between two groups with comparable variance were calculated by two-tailed Student's *t*-test. One-way ANOVA (between genotypes) was used for analysis between three groups with comparable variance followed by Bonferroni post-test for individual comparisons. Kaplan-Meier comparison was used for analysis of survival cohorts, and Fisher's exact test was used to analyze tumor grade data.

### Accession number

Microarray data were deposited in Gene Expression Omnibus as GSE94758.

## Supplementary Material

Supplemental Material
