# Supplementary material for: Lung tumors with distinct p53 mutations respond similarly to p53 targeted therapy but exhibit genotype-specific statin sensitivity
Source: Genes Dev. 2017 Jul 1;31(13):1339–53. doi: 10.1101/gad.298463.117 (PMC5580655; doi:10.1101/gad.298463.117)
Supplement: Supplemental Material [file supp_31_13_1339__index.html]

Lung tumors with distinct p53 mutations respond similarly to p53 targeted therapy but exhibit genotype-specific statin sensitivity — Supplemental Material 

# Lung tumors with distinct p53 mutations respond similarly to p53 targeted therapy but exhibit genotype-specific statin sensitivity

## Supplemental Material

- Supplemental\_Material.pdf
