# Supplementary material for: Lung tumors with distinct p53 mutations respond similarly to p53 targeted therapy but exhibit genotype-specific statin sensitivity
Source: Genes Dev. 2017 Jul 1;31(13):1339–53. doi: 10.1101/gad.298463.117 (PMC5580655; doi:10.1101/gad.298463.117)
Supplement: Supplemental Material [file supp_gad.298463.117_Supplemental_Material.pdf]

Supplemental_Fig_S1

A

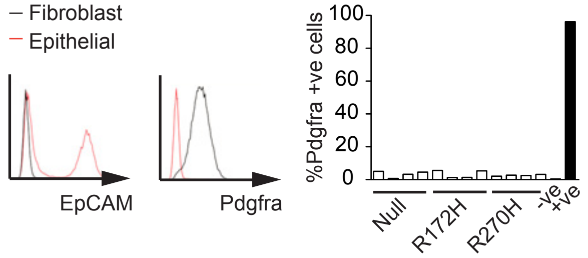

B

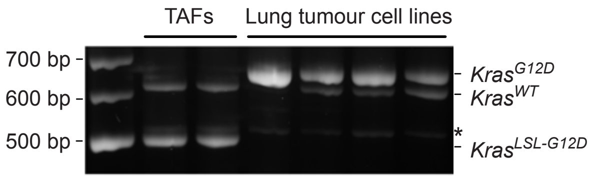

C

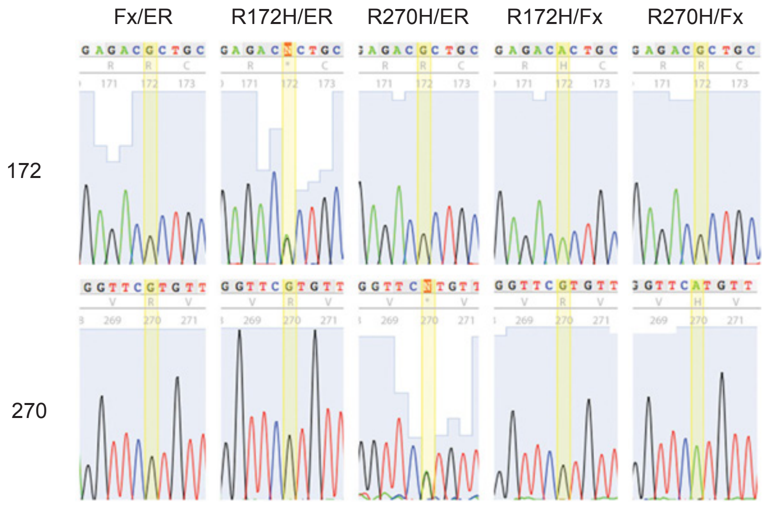

Codon	WT	Mutant
172	CGC	CAC
270	CGT	CAT

D

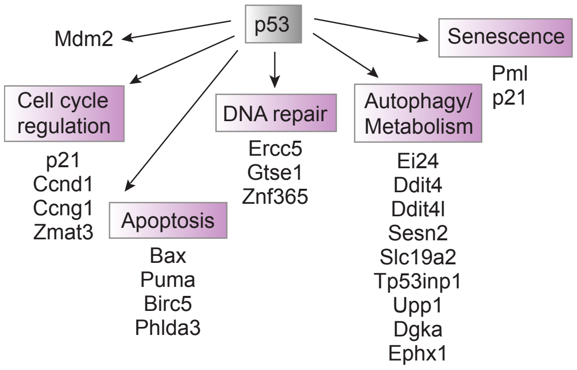

Supplemental Figure S1. Characterization of p53 null and mutant lung tumour cell lines and analysis of restored wild-type p53 signalling.

A. Left and middle, representative data showing EpCAM (epithelial marker; left) and Pdgfra (fibroblast marker; middle) expression in murine lung tumor cell lines and mouse embryonic fibroblasts (MEFs) based on FACS analysis. Right, percentage of Pdgfra-positive cells across the twelve tumor cell lines analyzed by microarray. Epithelial cells were used as a negative (-ve) and MEFs as positive (+ve) control. **B.** Genotyping of the KrasG12D allele (PCR) in lung tumor associated fibroblasts (TAFs) and tumor cell cultures derived from our murine models. Bands corresponding to the mutant unrecombined (KrasLSL-G12D), recombined (KrasG12D) or WT (KrasWT) alleles are indicated. (*) denotes a background band. **C.** Sequencing chromatograms illustrate the different p53 genotypes of murine tumor cell lines used. **D.** Diagram showing examples of p53 targets similarly regulated in all genotypes grouped according to their distinct cellular functions.

Supplemental_Fig_S2

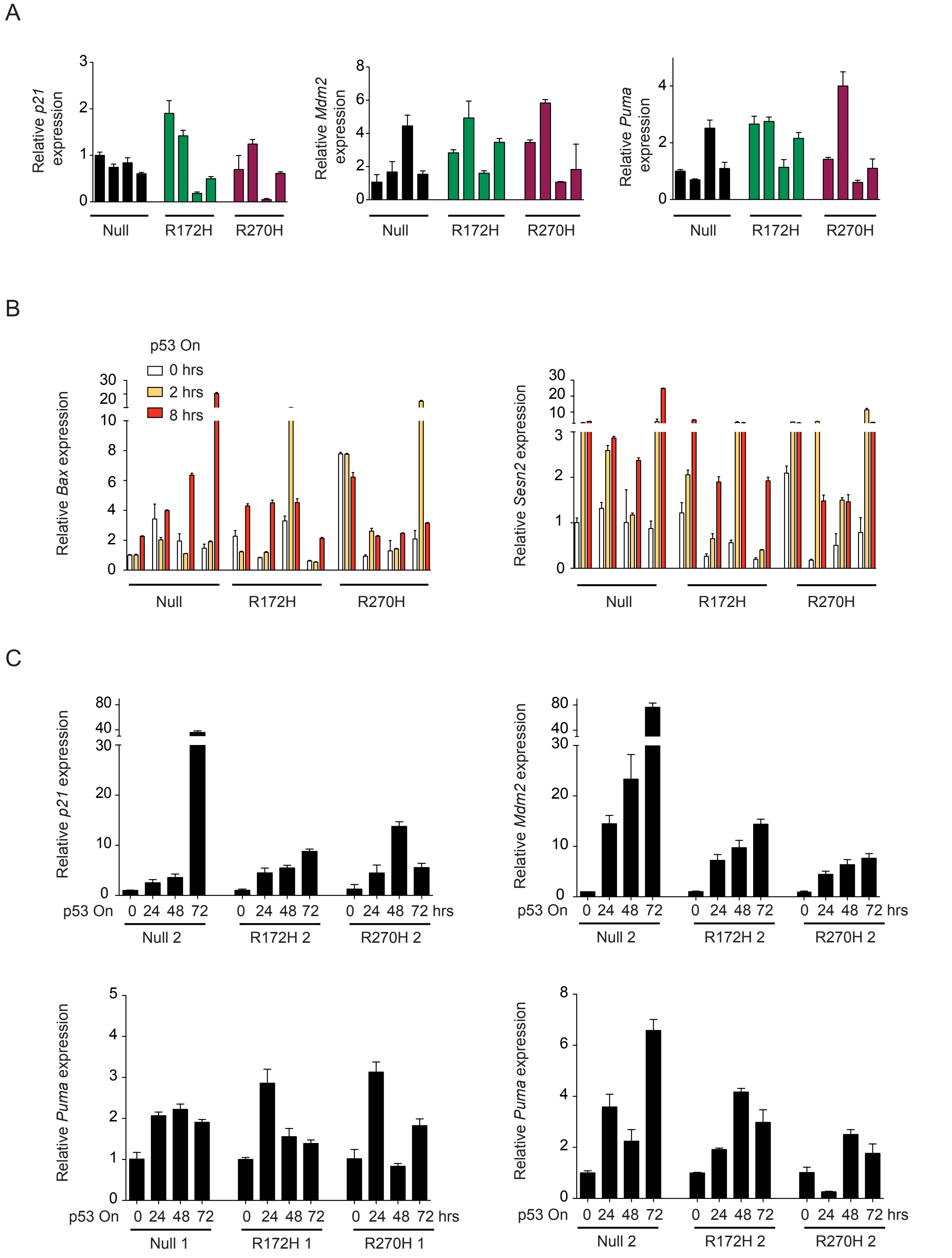

Supplemental Figure S2. WT p53 restoration induces key p53 target genes in p53 mutant and null cells.

Representative Taqman data (n=2 runs) show expression of genes in Null, R172H, R270H cell lines; SD of triplicate mean/cell line shown. **A.** Basal RNA expression of indicated genes in untreated cells shown relative to a Null sample (first bar). n=4 cell lines/genotype shown. **B, C** Expression of p53 target genes at indicated timepoints following vehicle/4OHT treatment. **B.** Expression relative to vehicle treated (p53 Off) Null cell line (first bar); n=4 cell lines/genotype. **C.** Expression shown relative to vehicle at corresponding timepoints; n=2 cell lines/genotype.

Supplemental_Fig_S3

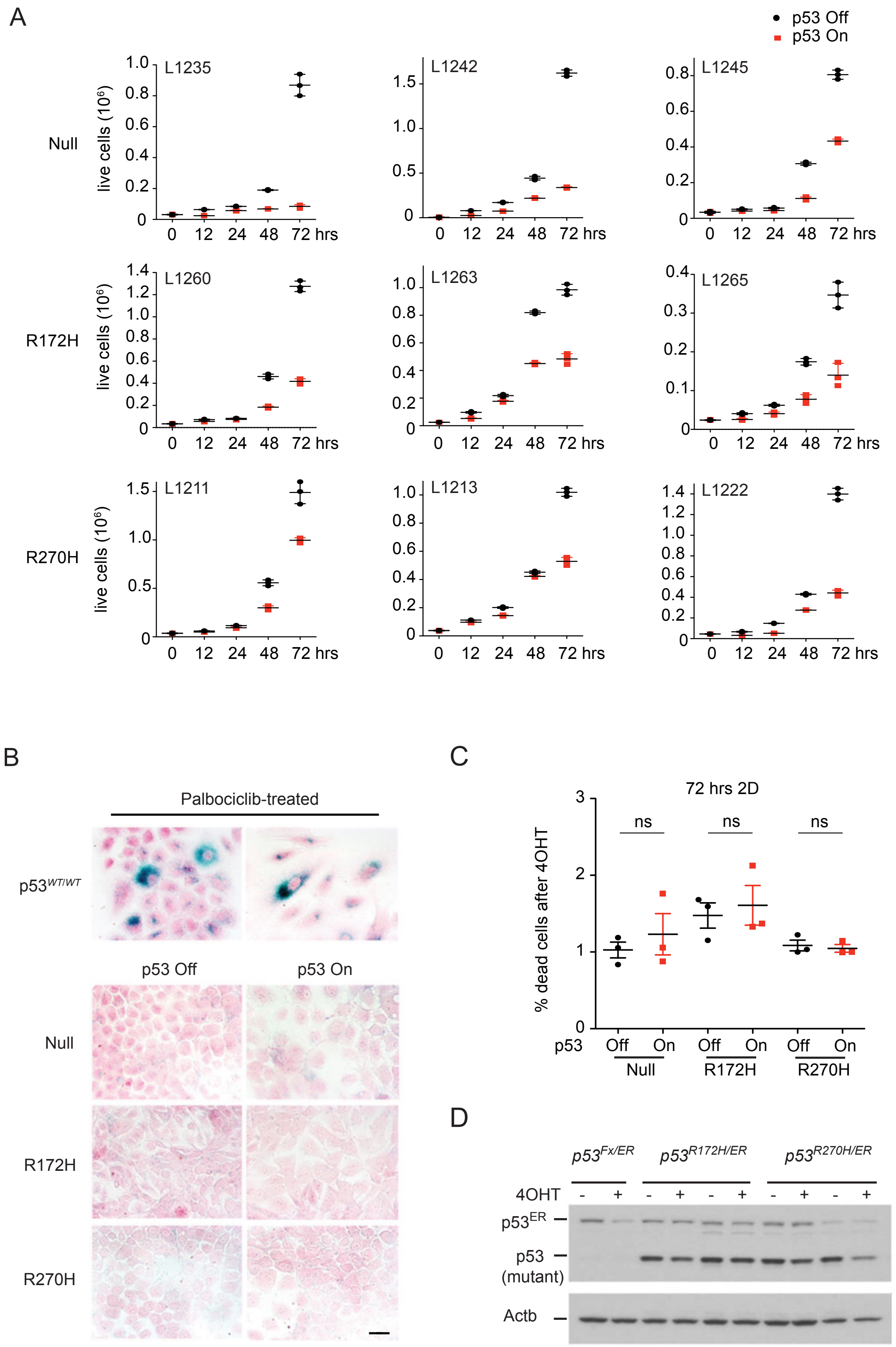

Supplemental Figure S3. Viability of Null, R172H and R270H lung tumor cell lines following p53 restoration.

A. Growth curves of p53 Null, R172H and R270H cell lines treated with vehicle (p53 Off) or 4OHT (p53 On). Three independent cell lines/genotype shown. Symbols denote technical triplicates and mean ±SD indicated. **B.** Representative β-galactosidase staining in p53 null and mutant cells (one cell line/genotype shown of two analysed) 72 hrs after p53 restoration. Two palbociclib-treated wild-type p53 human lung cancer cell lines shown as a positive control. Scale bar: 40 μm. **C.** Percentage dead cells 72 hrs after p53 restoration (2D cultures). Representative data (n=3 experiments) show one cell line/genotype ±SD of triplicates, *t*-test ns: non-significant. **D.** Expression levels of (mutant) p53, p53ER and Actb 24 hrs after Ctrl ("-") or 4OHT treatment ("+"), based on immunoblotting (see Figure 3H).

A

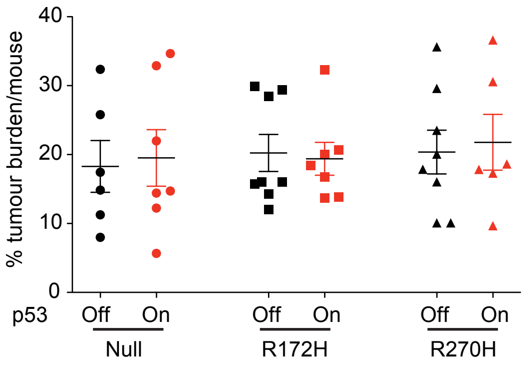

B

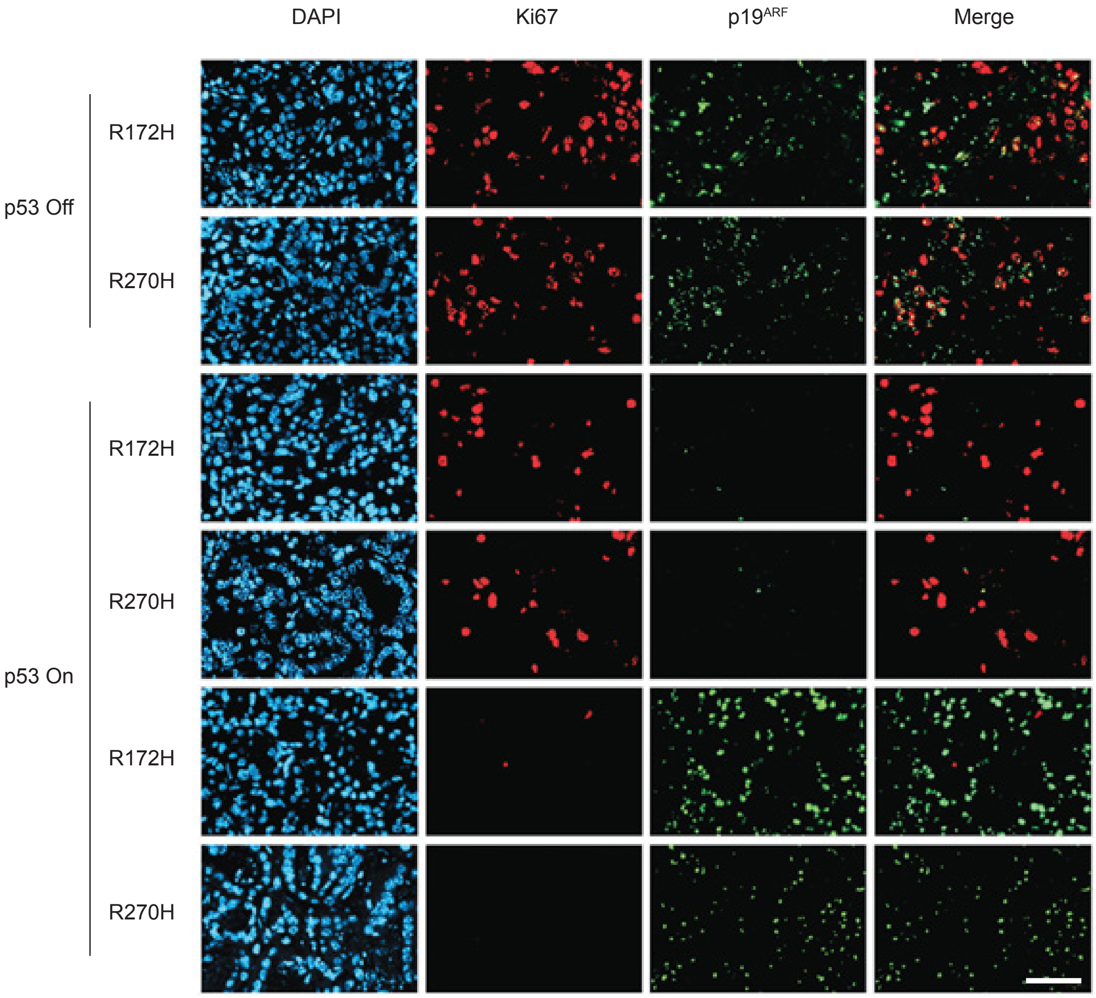

Supplemental Figure S4. Characterization of the effects of wild-type p53 restoration in p53 mutant and p53 null tumors.

A. Quantification of tumor burden/animal/genotype ±SEM (symbols represent individual mice) in the presence (p53 On) or absence (p53 Off) of p53 restoration (6 days). **B.** Representative data illustrates p19ARF (green) and Ki67 (red) expression in lung tumor sections in the presence or absence of p53 restoration (6 days). DAPI staining shown in blue. Scale bar: 60 μm.

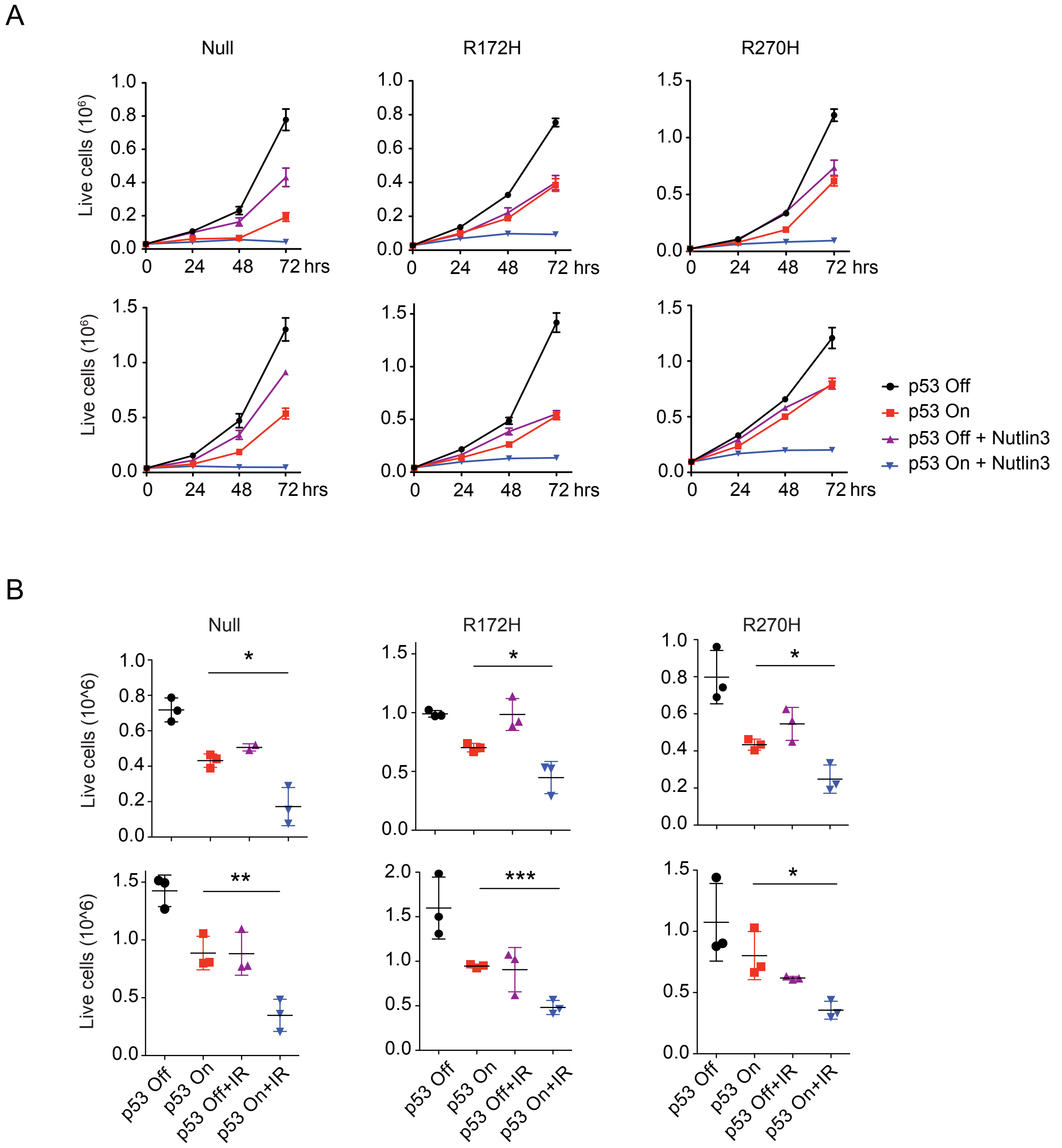

Supplemental Figure S5. p53 activation through exogenous signals enhances its tumor suppressive effect in p53-null and mutant lung tumor cells.

A. Growth curves for Null, R172H and R270H lung tumor cells treated with vehicle (p53 Off), 4OHT (p53 On), vehicle+Nutlin-3 or 4OHT+Nutlin-3. Representative data (n=3 experiments) shows two independent cell lines/genotype. Triplicate mean ±SD shown. **B.** Cell viability 48 hrs after exposure of indicated cells to vehicle, 4OHT, ionizing radiation (IR, 4Gy) or combined treatment (p53 On+IR). Representative data (n=3 experiments) show two independent cell lines/genotype (n=4). Symbols show values of triplicates and mean ±SD indicated, t-test; non-significant: ns,*P<0.05, **P<0.01, ***P<0.001.

Supplemental_Fig_S6

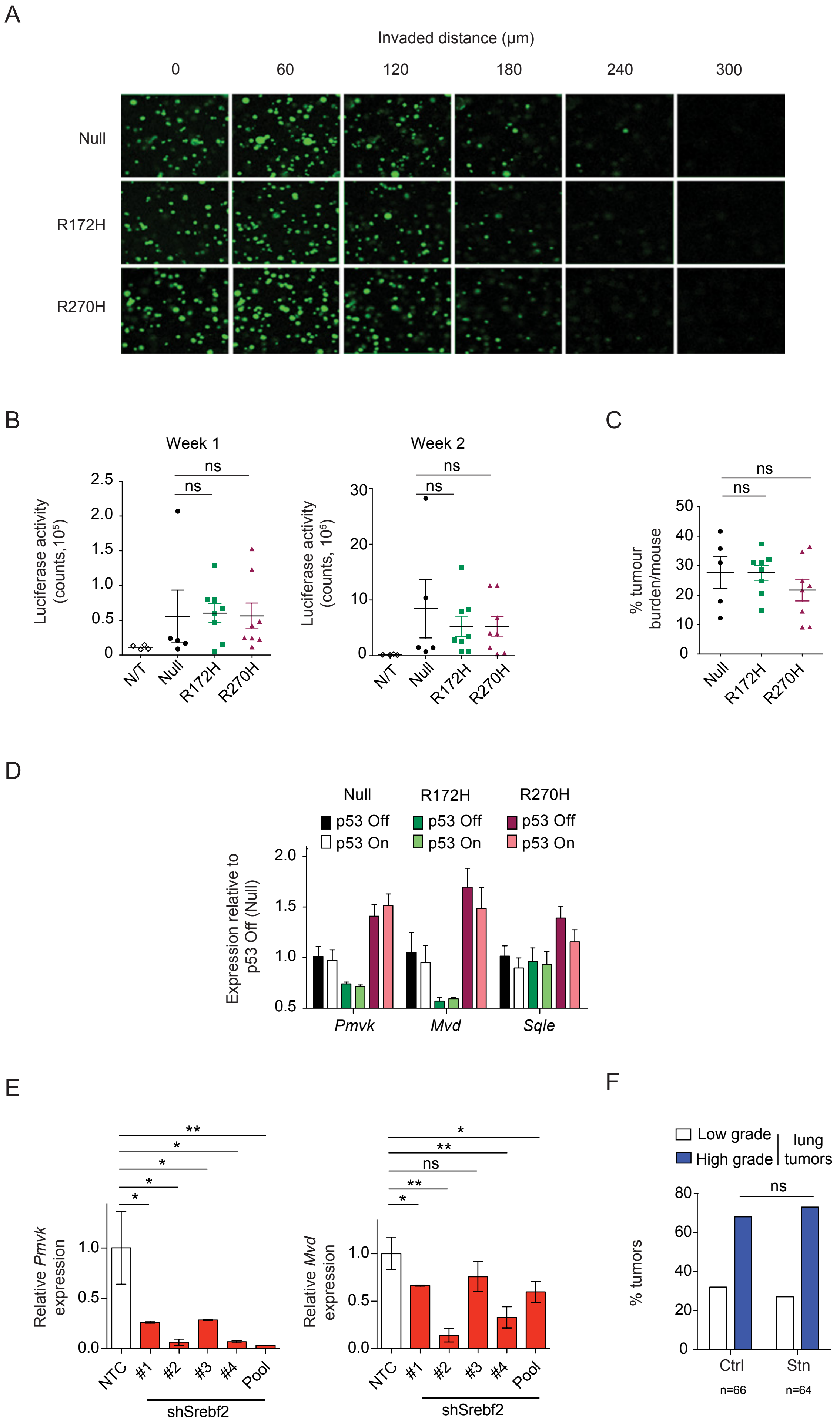

Supplemental Figure S6. Phenotypic characterization of p53 Null, R172H and R270H lung tumor cells.

A. Representative data (two cell lines examined/genotype) show invasive growth of p53-null and p53-mutant colonies through Matrigel in one cell line/genotype. Images of z-stack slices displaying invaded distance for each genotype (see Figure 5E). **B.** Luciferase activity of transplanted tumor cells of the indicated genotypes in the lungs of recipient mice one week (1) and two weeks (2) after transplantation (a minimum of 5 recipient mice transplanted/genotype). **C.** Percentage tumour burden/mouse/genotype at end point for mice shown in (B). **D.** Expression of Pmvk, Mvd and Sqle in Null, R172H and R270H cells following vehicle treatment (Off) or 8 hrs of p53 restoration (On). Average expression (n=4 cell lines/genotype) relative to Null cells (Off) shown ±SEM (microarray data). **E.** Representative Taqman data (n=2 independent runs) for indicated genes in R270H cells after Srebf2 knockdown (3D cultures). SD of triplicate mean/cell line shown. NTC, non-targeting control; Pool = all four shRNAs. (see Figure 6F). **F.** Quantification of low- and high-grade tumor frequencies in R270H mice treated with Stn for 6 days. A minimum of 3 animals/cohort were analysed, ns: non-significant, Fischer's Exact Test.

Supplemental Table S1. Genes similarly regulated by p53 in Null, R172H and R270H cells

	Gene	Target gene evidence			Gene	Target gene evidence	
		ChIP*	Functional**			ChIP*	Functional**
2 hrs	Slc19a2	Yes	Yes	8 hrs	Blcap	Yes	No
	Bbc3	Yes	Yes		Mast4	Yes	No
	Mdm2	Yes	Yes		Crip2	Yes	No
	Cdkn1a	Yes	Yes		Syncrip	Yes	No
	Tp53inp1	Yes	Yes		Eprs	Yes	No
	Sesn2	Yes	Yes		Ccdc3	Yes	No
	Ccng1	Yes	Yes		Ckap2	Yes	No
	Znf365	Yes	Yes		Nfe2l2	Yes	No
	Phlda3	Yes	Yes		Areg	Yes	No
	Ddit4l	No	Yes		Rnf169	Yes	No
	Gtse1	No	Yes		Hist1h2ab	Yes	No
8 hrs	Birc5	Yes	Yes		Mmp14	Yes	No
	Zmat3	Yes	Yes		Hist1h2ah	Yes	No
	Dgka	Yes	Yes		Hist1h2ae	Yes	No
	Ercc5	Yes	Yes		Slc30a1	Yes	No
	Upp1	Yes	Yes		Slc4a11	Yes	No
	Pml	Yes	Yes		Ephx1	No	Yes
	Ddit4	Yes	Yes		Ivl	No	Yes
	Ccnd1	Yes	Yes		Itgb4	No	Yes
	Apaf1	Yes	Yes		Ei24	No	Yes
	Bax	Yes	Yes		Hist1h2aa	No	No
	Mcm3	Yes	No		Aunip	No	No
	Evpl	Yes	No		Clspn	No	No
	Top2a	Yes	No		Kiaa0101	No	No
	Sh3bgrl2	Yes	No		Mcm10	No	No
	Klhl26	Yes	No		Hist1h2aj	No	No
	Esco2	Yes	No		Hist1h2am	No	No
	Nek3	Yes	No		Hist1h2ap	No	No
	Pbk	Yes	No		Gdpd5	No	No
	Kif15	Yes	No		Ggta1	No	No
	Irf2bp1	Yes	No		Aldh4a1	No	No
	Pgpep1	Yes	No		Prr15l	No	No
	Plau	Yes	No		St3gal3	No	No
	St3gal2	Yes	No		Kank3	No	No
	B3galnt2	Yes	No		Anxa8	No	No
	Commd3	Yes	No		Bscl2	No	No
	Cpped1	Yes	No		Tmem150a	No	No
	Inf2	Yes	No		Glipr1	No	No
	Dyrk3	Yes	No		Hes6	No	No
	Trim11	Yes	No		Fam2l2b	No	No
	Dcxr	Yes	No		Hya1	No	No
	Chaf1b	Yes	No		Prkd2	No	No
	Gsto1	Yes	No		Tmem19	No	No
	Nrm	Yes	No				

* Genes identified as p53 targets based on ChIP data (http://chip-atlas.org and Fischer et al., 2017). **Genes identified as p53 targets based on functional studies (GSEA; Okamoto and Beach 1994; Lehar et al. 1996; Utrera et al. 1998; Bieging et al. 2014; Lo et al. 2001; Quintens et al. 2015).

Supplemental Table S2. Distribution of direct p53 targets across different p53 restoration response groups.

		R172H		R270H	
		No. of genes	Direct target (%)*	No. of genes	Direct target (%)*
Commonly regulated	2 hrs	11	82	11	82
	8 hrs	76	64	76	64
	WT-like	87	71	43	70
Dominant negative	Decreased ability	143	55	155	55
	Complete failure	72	43	27	41

*Genes identified as direct p53 targets based on ChIP data (Fischer et al., 2017 and http://chip-atlas.org).

Supplemental Table S3. Classification of *TP53* mutations in lung adenocarcinoma cases (TCGA, Nature 2014)

TCGA ID	*TP53* Mutation	Mutation class
TCGA-50-6595-01	R175H, S183*	conformational
TCGA-91-6840-01	Y220C	conformational
TCGA-78-7536-01	F270C	conformational
TCGA-97-7553-01	R282W	conformational
TCGA-44-6777-01	G245V	conformational
TCGA-97-7554-01	R249W	conformational
TCGA-44-6775-01	H168L	conformational
TCGA-05-4430-01	R249M	conformational
TCGA-64-1679-01	G245V	conformational
TCGA-69-7760-01	F270V	conformational
TCGA-05-5423-01	R249M,I195M	conformational
TCGA-44-6774-01	G245C	conformational
TCGA-49-4514-01	R175H	conformational
TCGA-75-6214-01	V157F	conformational
TCGA-44-2662-01	R280T	contact
TCGA-05-4432-01	D281E, R342*	contact
TCGA-78-7149-01	R248L	contact
TCGA-05-4410-01	D281V	contact
TCGA-38-6178-01	R283P	contact
TCGA-55-1596-01	D281Y	contact
TCGA-05-4420-01	R280I	contact
TCGA-55-7724-01	R273H	contact
TCGA-64-5775-01	R280G	contact
TCGA-78-7540-01	R273C	contact
TCGA-95-7039-01	R273L	contact
TCGA-49-4505-01	C277F	contact
TCGA-05-4405-01	R175H, C277F	both

TP53 mutations classified as conformational or contact based on effects on the protein structure or residues in direct contact with DNA according to Joerger et al, 2006; Joerger and Fersht, 2007; Cho et al, 1994.
